# Agglutinin-Like Sequence (*ALS*) Genes in the *Candida parapsilosis* Species Complex: Blurring the Boundaries Between Gene Families That Encode Cell-Wall Proteins

**DOI:** 10.3389/fmicb.2019.00781

**Published:** 2019-04-26

**Authors:** Soon-Hwan Oh, Brooke Smith, Andrew N. Miller, Bart Staker, Christopher Fields, Alvaro Hernandez, Lois L. Hoyer

**Affiliations:** ^1^Department of Pathobiology, University of Illinois at Urbana-Champaign, Urbana, IL, United States; ^2^Department of Biology, Millikin University, Decatur, IL, United States; ^3^Illinois Natural History Survey, Urbana, IL, United States; ^4^Seattle Structural Genomics Center for Infectious Disease, Seattle Children’s Hospital, Seattle, WA, United States; ^5^Roy J. Carver Biotechnology Center, University of Illinois at Urbana-Champaign, Urbana, IL, United States

**Keywords:** agglutinin-like sequence genes, *ALS* family, *Candida* species, adhesion, cell-wall proteins, fungi

## Abstract

The agglutinin-like sequence (Als) proteins are best-characterized in *Candida albicans* and known for their role in adhesion of the fungal cell to host and abiotic surfaces. *ALS* sequences are often misassembled in whole-genome sequence data because each species has multiple *ALS* loci that contain similar sequences, most notably tandem copies of highly conserved repeated sequences. The *Candida parapsilosis* species complex includes *Candida parapsilosis*, *Candida orthopsilosis*, and *Candida metapsilosis*, three distinct but closely related species. Using publicly available genome resources, *de novo* genome assemblies, and laboratory experimentation including Sanger sequencing, five *ALS* genes were characterized in *C. parapsilosis* strain CDC317, three in *C. orthopsilosis* strain 90–125, and four in *C. metapsilosis* strain ATCC 96143. The newly characterized *ALS* genes shared similar features with the well-known *C. albicans ALS* family, but also displayed unique attributes such as novel short, imperfect repeat sequences that were found in other genes encoding fungal cell-wall proteins. Evidence of recombination between *ALS* sequences and other genes was most obvious in *CmALS2265*, which had the 5′ end of an *ALS* gene and the repeated sequences and 3′ end from the *IFF/HYR* family. Together, these results blur the boundaries between the fungal cell-wall families that were defined in *C. albicans*. TaqMan assays were used to quantify relative expression for each *ALS* gene. Some measurements were complicated by the assay location within the *ALS* gene. Considerable variation was noted in relative gene expression for isolates of the same species. Overall, however, there was a trend toward higher relative gene expression in saturated cultures rather than younger cultures. This work provides a complete description of the *ALS* genes in the *C. parapsilosis* species complex and a toolkit that promotes further investigations into the role of the Als proteins in host-fungal interactions.

## Introduction

The study of adhesive mechanisms is pivotal in pathogenic microbiology because adhesion promotes host colonization, which is a foothold in the process of causing disease. The agglutinin-like sequence (*ALS*) gene family encodes cell-surface glycoproteins that are involved in adhesion of fungal cells to host and abiotic surfaces (Hoyer and Cota, 2016). *ALS* genes and their encoded proteins are best characterized in *C. albicans*. A binding cavity located within the N-terminal Als domain is responsible for adhesion to peptide ligands (Lin et al., 2014). Another hallmark of *C. albicans* Als proteins is an often-extensive central domain of tandemly repeated sequences that are rich in serine, threonine, and sometimes proline.

The rapid pace at which draft genome sequences are developed is a benefit to the research community. At this time, however, most available draft genomes were assembled from short-read DNA sequences of no more than a few hundred nucleotides (e.g., Riccombeni et al., 2012; Pryszcz et al., 2015). Considering that the repeated DNA unit in *ALS* genes is 108 bp and that the tandemly repeated copies of highly similar sequences may extend for several kilobases, it is no surprise that many genome assemblies fail within the *ALS* coding regions. In some published fungal genomes, *ALS* genes may be assembled accurately, while in others, it is not possible to calculate basic information such as the number of loci present in the gene family. Long-read DNA sequence technology provides the opportunity to create a larger template upon which the more-accurate short-read sequences can be assembled (Madoui et al., 2015), providing a new approach to characterizing the *ALS* genes in pathogenic fungi.

The focus of this work is the *Candida parapsilosis* species complex: *Candida parapsilosis*, *Candida orthopsilosis*, and *Candida metapsilosis*. The three species are closely related; recognition as separate species is a relatively recent event (Tavanti et al., 2005). The species are gaining importance as pathogens, with adhesion and colonization mechanisms playing a central role in the disease process (Bliss, 2015). As such, it is important to understand the nature of the *ALS* family, its variability across alleles within and among various strains, and the relative gene expression patterns that may reveal which of the genes could play the largest role in the adhesion process.

Using a combination of published sequence data and *de novo* approaches, we establish the nature of the *ALS* genes in the *C. parapsilosis* species complex, their allelic variability, and expression patterns. The resulting data reveal novel sequences and recombination between genes that demonstrate the blurring of gene family definitions that were developed in *C. albicans*. The work provides a toolkit that can be used to further knowledge of the *ALS* genes, as well as highlights mechanisms for maintaining the diversity of cell-surface adhesins in the pathogenic fungi.

## Materials and Methods

### Microbial Strains

Table 1 shows the microbial strains used in this study. Fungal identifications were validated using RAPD methodology (Tavanti et al., 2005). Products were separated on 1% agarose/Tris-Acetate-EDTA gels and visualized by ethidium bromide staining. Routine PCR analyses were conducted as described previously (Green et al., 2004).

**Table 1:** *C. parapsilosis*, *C. orthopsilosis*, and *C. metapsilosis* strains and sources.

Species	Strain	Source
*Candida parapsilosis*	CDC317 (ATCC MYA-4646)	Geraldine Butler (UC Dublin)
	949 (44)	Patricia Kammeyer (Loyola)
	950 (X36406)	Patricia Kammeyer (Loyola)
	1125 (ATCC 22019)	American Type Culture Collection
*Candida orthopsilosis*	90–125	Arianna Tavanti (Pisa)
*Candida metapsilosis*	ATCC 96143	Arianna Tavanti (Pisa)
	61	Arianna Tavanti (Pisa)
	88	Arianna Tavanti (Pisa)
	397	Arianna Tavanti (Pisa)
	482	Arianna Tavanti (Pisa)

### Publicly Available Genome Sequences

Previously published genome sequences were used for this study. These included *C. parapsilosis* strains CDC317 (Butler et al., 2009; GenBank accession number ASM18276v2; GCA_000182765.2), CBS6318 (GCA_0000982555.2), and GA1 (GCA_0000982675.1). Strain CDC317 was used as the representative genome sequence. A genome sequence was available for *C. orthopsilosis* strain 90-125 (GCA_000315875.1). A new 90–125 sequence was derived from short-read and long-read sequence data as described previously (Lombardi et al., 2019; PQBP00000000). Sequences for strains MCO456 (GCA_900002835.2) and AY2 (GCA_000304155.1) were also examined. The *C. metapsilosis* genome sequence under accession number CBZN0200000000 was included in the analysis although it was compiled from more than one isolate (Pryszcz et al., 2015). A new *C. metapsilosis* genome sequence was generated from short-read and long-read sequence data as described below.

### Genome Sequence Data Collection, Assembly, and Validation

A new genome sequence was generated for *C. metapsilosis* strain ATCC 96143. Genomic DNA initially was extracted from cells that were grown to saturation (16 h) at 37°C in YPD medium (per liter: 10 g yeast extract, 20 g peptone, 20 g glucose) with 200 rpm shaking. The genome assembly from this preparation was of lower quality than desired, so the method was repeated, this time with a culture that was grown to mid-log phase. Mid-log-phase cells appeared to lack an extracellular polysaccharide that was present in the saturated culture, presumably leading to a cleaner DNA preparation that did not clog the pores of the MinION sequencer (see below). The mid-log phase sequence data replaced those from the saturated culture prep in the GenBank deposit (PQNC00000000) and were used in the analysis presented here.

DNA was extracted using the method of Sherman et al. (1986). Briefly, cells were treated with zymolyase (MP Biomedicals) to form spheroplasts that were lysed with sodium dodecyl sulfate. Gentle mixing by inversion was used to handle the spheroplasts, and during subsequent phenol extraction and isopropyl alcohol precipitation of the DNA. Verification of the quality of the high-molecular-weight DNA was assessed using agarose gel electrophoresis.

*C. metapsilosis* DNA libraries were constructed and sequenced at the Roy J. Carver Biotechnology Center, University of Illinois at Urbana-Champaign. Data were derived using Illumina (short-read) and Oxford Nanopore (long-read) methods. MiSeq shotgun genomic libraries were prepared with the Hyper Library construction kit (Kapa Biosystems). The library was quantitated by qPCR and sequenced on one MiSeq flowcell for 151 cycles from each end of the fragment using a MiSeq 300-cycle sequencing kit (version 2). FASTQ files were generated and demultiplexed with the bcl2fastq Conversion Software (Illumina, version 2.17.1.14). MiSeq reads were quality trimmed using Trimmomatic (Bolger et al., 2014) with the parameters “LEADING:30 TRAILING:30” prior to assembly.

For Oxford Nanopore long-read sequencing, 1 μg of genomic DNA was sheared in a gTube (Covaris, Woburn, MA, United States) for 1 min at 6,000 rpm in a MiniSpin plus microcentrifuge (Eppendorf, Hauppauge, NY, United States). The sheared DNA was converted into a shotgun library with the LSK-108 kit from Oxford Nanopore, following their manual. The library was sequenced on a SpotON R9.4 RevC flowcell for 48 h using a MinION MK 1B sequencer.

Basecalling was performed with the software Albacore version 2.3.1 from Oxford Nanopore. Sixty nucleotides were removed from both ends of each Oxford Nanopore read, followed by additional trimming using a Github checkout (commit 92c0b65f) of Porechop (Wick et al., 2017a) to remove reads with potential internal barcodes which were likely chimeric. Only reads longer than 800 nt were used in the final assembly. Canu v1.7 (Koren et al., 2017) was used for assembly with the following parameters: ‘canu -p asm -d C_meta_default genomeSize = 14m useGrid = false -nanopore-raw c_metapsilosis.qualtrim.clean.fastq.gz.’

Oxford Nanopore reads were then aligned against the assembly using minimap v2.8 (Li, 2018), and the alignment was then used to polish the assembly using nanopolish v 0.9.0 (Senol Cali et al., 2018). Quality-trimmed MiSeq data were used to iteratively polish the assembly using a Python script based on the iterative polishing method utilized during assembly with the hydrid assembler Unicycler (Wick et al., 2017b) using Pilon v1.22 for error correction (Walker et al., 2014). Haplomerger2 (release 20180603; Huang et al., 2017) was then used to both locate and resolve potential haplotypes, generating a haploid reference genome with a separate assembly with alternate haplotypes.

Ambiguities in the genome sequence data were corrected by PCR amplification of the region and Sanger sequencing of the product. Supplementary Table S1 lists PCR primer sequences. Supplementary File S1 includes details regarding the computational analyses and characteristics of the resulting *C. metapsilosis* genome sequence.

### Tools for Identification of *ALS* Genes and Features of Predicted Als Proteins

BLAST^1^ was used to identify potential *ALS* genes and Als proteins in the various genome sequences. *C. albicans* sequences used as queries included *CaALS1* (L25902), *CaALS2* (AH006927), *CaALS3* (AY223552), *CaALS4* (AH006929), *CaALS5* (AY227440), *CaALS6* (AY225310), *CaALS7* (AF201684), *CaALS9-1* (AY269423), and *CaALS9-2* (AY269422). As additional *ALS* genes were located, those sequences were also used as BLAST queries until no additional new sequences were detected.

Secretory signal peptides were detected in putative Als proteins using the SignalP 4.1 Server^2^ (Nielsen, 2017). Putative GPI anchor addition sites were identified using the big-PI Predictor^3^ (Eisenhaber et al., 1999). Nucleotide sequence translation, multiple sequence alignments, and other general bioinformatic processes used the European Bioinformatics Institute (EMBL-EBI) tools and data resources^4^ (Cook et al., 2017).

### Phylogenetics Analysis

Nucleotide sequences from 21 concatenated genes from the *ALS* family were analyzed using maximum likelihood (ML) and Bayesian methods. Alignment of genes was conducted in SeaView v4.6.1 (Gouy et al., 2010) using Muscle v3.8 (Edgar, 2004). Ambiguous regions were removed using Gblocks v0.91b (Castresana, 2000), allowing for less strict flanking regions, gap positions within the final blocks, and smaller final blocks. Maximum likelihood analysis was conducted using PhyML (Guindon and Gascuel, 2003) under the GTR substitution model with four rate classes and optimized invariable sites based on the results from jModelTest 2.0 (Posada, 2008). The best nearest neighbor interchange and subtree pruning and regrafting tree improvement was implemented on the unrooted BioNJ starting tree and branch support was determined with 1,000 non-parametric bootstrap replicates. Bayesian analysis was conducted using MrBayes 3.2.2 on XSEDE (3.2.6) through the CIPRES Science Gateway (Miller et al., 2010). Bayesian analysis was conducted using the GTR + I + G model with four rate classes and four independent chains and ran for 10 million generations sampling every 1,000 trees with the first 25% of trees discarded as burn-in. A consensus tree with the remaining 7,500 trees was produced using PAUP 4.0a (Swofford, 2002). Nodes with ≥70% bootstrap support and ≥95% Bayesian posterior probability were considered significantly supported (Alfaro et al., 2003).

### Structural Analysis

Structural homology models were calculated using Phyre2 (Kelly et al., 2015). Sequence alignments were created to identify structurally conserved residues and models were built using the Threading option to maintain the integrity of the peptide-binding cavity (PBC) and allow for comparison of side-chain residue changes between sequence variants. Structures were compared and analyzed using Coot (Emsley et al., 2010) and Maestro (Schrodinger LLC, Portland, OR, United States).

### Growth Conditions for Gene Expression Analysis

All fungal isolates were stored as pure cultures in 30% glycerol at -80°C. Isolates were streaked to YPD agar as needed. YPD plates were stored at 4°C for no more than 1 week prior to use. Some growth conditions were used for assessing *ALS* gene expression because they mimicked conditions used in another publication. Other selected conditions were intended to detect large trends in differential gene expression that are characteristic of the *C. albicans ALS* family (e.g., assessing gene expression at early or late time points in different growth media; Hoyer et al., 2008). In each experiment, cells were collected from the specified growth condition, pelleted by centrifugation, and the pellet flash frozen in a dry ice-ethanol bath. Frozen pellets were stored at -80°C until RNA was extracted.

*C. orthopsilosis* strain 90-125 was grown to mimic the conditions described by Lombardi et al. (2019). A single colony from a YPD plate was inoculated into 10 ml YPD liquid medium and incubated for 24 h at 30°C and 200 rpm shaking. 0.5 ml of the culture was inoculated into 20 ml fresh YPD medium and incubated for either 1 h or 24 h under the same temperature and agitation conditions. Cells were harvested by centrifugation, flash frozen in dry ice/ethanol, and stored at -80°C. Duplicate cultures were grown on three separate occasions.

A similar approach was used for gene expression analysis of the *C. parapsilosis* genome isolate (CDC317) and the type strain (ATCC 22019). A single colony from a YPD plate was inoculated into 10 ml YPD medium and grown for 24 h at 30°C and 200 rpm shaking. *C. parapsilosis* cells were also grown using the liquid filament induction conditions of Lackey et al. (2013). In preliminary experiments, growth of strain ATCC 22019 for 24 h produced slightly elongated cells while strain CDC317 appeared as a typical budding yeast. One colony was inoculated into 20 ml YPD at 30°C and 200 rpm shaking for 24 h. Cells were pelleted by centrifugation and washed twice in sterile water. A culture of SC medium [6.7 g/liter yeast nitrogen base without amino acids, supplemented 2% glucose and 2 g of complete amino acid mixture as defined by Lackey et al. (2013)] containing 20% fetal bovine serum (Gibco; 26140-079) was inoculated at an OD_600_ of 0.6. The culture was incubated for 2 h at 37°C and 200 rpm shaking. Duplicate cultures were grown on three separate occasions.

For *C. metapsilosis*, genome strain (ATCC 96143) and strains 61, 397, and 482 were studied. A single colony from the YPD plate was inoculated into 10 ml YPD liquid medium and incubated for 24 h at 37°C and 200 rpm shaking. Cells were harvested from the 24-h culture, replicate 2.5-ml aliquots were removed from the culture; cells were collected, frozen, and stored as described above. The remaining 5 ml of culture was washed in Dulbecco’s Phosphate Buffered Saline (BioWhittaker; 17-512Q), diluted, and counted using a hemocytometer. The total cell number was resuspended in RPMI 1640 tissue culture medium (Gibco; 11875-135) at a density of 5 × 10^7^ cells/ml. The culture was incubated for 1 h at 37°C and 200 rpm shaking. Cells were collected by filtration across a 0.45-micron membrane (GVS Life Sciences; 1213776). The filter was placed into a 50-ml conical centrifuge tube, flash frozen, and stored at -80°C.

### Design and Validation of TaqMan Assays to Quantify Relative *ALS* Gene Expression Levels

All steps for developing, validating, and running the TaqMan assays followed the Guide to Performing Relative Quantitation of Gene Expression Using Real-Time Quantitative PCR (Applied Biosystems Inc., 2008). TaqMan primers and probes were designed using PrimerQuest (Integrated DNA Technologies). Primers had an optimal *T*_m_ of 61°C, GC content of 40% and 25-nt length. Probes had an optimal *T*_m_ of 70°C, GC content of 40% and 28-nt length. To identify divergent sequences that could be exploited for TaqMan assay design, Clustal Omega^5^ was used to align the nucleotide sequences from the 5′ end of each *ALS* gene. An amplicon size of 140 bp was sought; some products were larger or smaller, depending on the availability of unique sequences among the genes. PCR efficiencies were measured for each TaqMan assay; each was between 90 and 110% (Supplementary Table S2).

Because some genes were so similar within a given species, specificity of the TaqMan assays was demonstrated experimentally (Supplementary Table S3). Fragments were PCR amplified and cloned into the pJet1.2/blunt vector and transformed into *E. coli* INVαF’ (Thermo Fisher). The accuracy of cloned fragments was verified using Sanger sequencing (Roy J. Carver Biotechnology Center, Urbana, IL, United States). Dilutions of purified, quantified plasmid DNA were used as templates to demonstrate specificity of TaqMan assays (see section “Results”).

Control TaqMan assays featured the *ACT1* and *TEF1* genes from each species. Primers (Supplementary Table S1) were used to amplify the genes from each species and validate its sequence using the Sanger method. Some TaqMan control assays were designed to recognize a single species (Supplementary Table S2). Control assays that recognize multiple species were also designed with an eye on cost savings, as use of a common control would eliminate the need to order one control assay per species.

### Real-Time PCR Analysis of Gene Expression

RNA was extracted using the hot acidic phenol method of Collart and Oliviero (1993) with the addition of approximately 100 μl volume of 0.5 mm sterile, baked, acid-washed glass beads to each tube. Nucleic acid concentration was measured using a NanoDrop spectrophotometer (Thermo Scientific). The presence of contaminating genomic DNA in RNA preparations was detected by PCR using ITS4 and ITS5 primers^6^ (White et al., 1990). The reaction template was 25 ng of RNA in a 25 μl reaction containing PCR buffer with 2.5 mM of MgCl_2_, 1 μM of each primer, 0.5 mM of dNTP, and Taq polymerase (Invitrogen; 10342-020). PCR conditions were 93°C for 5 min followed by 40 cycles of 93°C for 30 s, 53°C for 30 s, 72°C for 1 min with a 7-min final extension at 72°C. DNase (Invitrogen; AM2224) was used according to manufacturer’s instructions to digest contaminating genomic DNA. Often, multiple rounds of DNase digestion were required to eliminate DNA below the detection limit of the PCR assay. Each RNA sample was cleaned using an RNeasy Mini spin column (Qiagen; 74104). RNA preparations were quantified and their quality assessed using Qubit (Invitrogen; Q10210) and/or the 2100 Bioanalyzer (Agilent Technologies). Aliquots were stored at -80°C until used for real-time gene expression assays.

The Superscript III cDNA synthesis kit (Invitrogen; 18080-051) was used to make cDNA according to manufacturer’s instructions. The amount of RNA added to the cDNA synthesis reaction was titrated by adding RNA dilutions to the cDNA synthesis reaction, then using the reaction products as the basis for real-time PCR quantification of a control gene. The C_t_ value for 0.2 μg RNA was in the linear portion of the graph of RNA amount vs. C_t_, so 0.2 μg RNA was used for all cDNA synthesis reactions.

The TaqMan assay reaction mixture was 15 μl in PrimeTime Gene Expression Master Mix (Integrated DNA Technologies) with 500 nM of each primer and 250 nM of probe. Synthesized cDNA was diluted 1:5 and 5 μl added to each reaction. PCR amplification used an ABI 7500 Real-Time PCR System (Applied Biosystems) with 95°C for 3 min followed by 40 cycles of 95°C for 15 s and 60°C for 1 min. Triplicate reactions were run for the *C. metapsilosis* RNA samples while duplicate reactions were run for the other species to fit as many reactions from the same species onto a single PCR plate.

Combining data across plates relied on an identical set of controls that were present on each: CmACT1 and CmTEF1 for *C. metapsilosis* RNA, and CmCoCpACT1 and CmCoCpTEF1 for *C. parapsilosis* and *C. orthopsilosis* samples. TaqMan analyses for comparison to SYBR Green data used only the CmCoCpACT1 control since the published SYBR Green assay relied only on *ACT1* as a control (Lombardi et al., 2019). The default threshold for each primer was applied and ΔC_t_ calculated using Expression Suite software v1.1 (Thermo Fisher Scientific).

A control sample of RNA without reverse transcriptase was analyzed using the CmCoCpACT1 TaqMan assay to monitor background amplification caused by residual genomic DNA. In all reactions but one (*C. metapsilosis* 1-Y-61; C_t_ = 38.7), no detectable product was generated during 40 amplification cycles.

Simple calculation of means and standard errors of the mean used Prism7 software (GraphPad). The statistical significance of results was assessed using a mixed-model analysis of variance (PROC MIXED in SAS 9.4; SAS Institute Inc., Cary, NC, United States). Separation of means used the LSMEANS option.

## Results

### Number and Nature of *ALS* Genes in Each Species of the *C. parapsilosis* Complex

**FIGURE 1:**
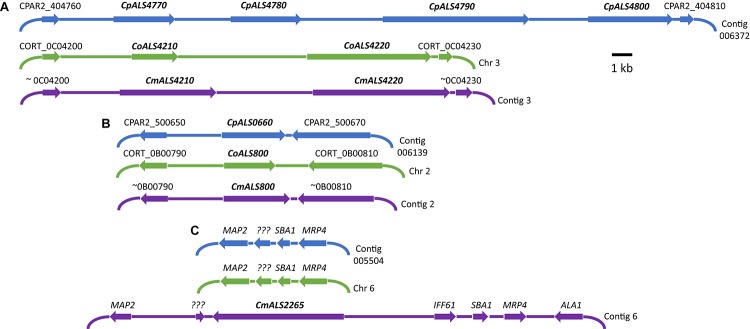
Schematic of chromosomal locations of *ALS* genes in *C. parapsilosis*, *C. orthopsilosis*, and *C. metapsilosis*, drawn to scale. **(A)** Location of contiguous *ALS* genes on *C. parapsilosis* contig 006372, *C. orthopsilosis* chromosome 3, and contig 3 from the *C. metapsilosis* genome assembly PQNC00000000. *ALS* gene names are in bold italics type. Arrows show the direction of transcription for each gene. Flanking sequences were identified to indicate gene order conservation among the three species. Names of the flanking ORFs were taken from the *Candida* Genome Database (www.candidagenome.org) and/or GenBank. **(B)** Location of genes *CpALS0660* (*C. parapsilosis* contig 006139), *CoALS800* (*C. orthopsilosis* chromosome 2), and *CmALS800* (*C. metapsilosis* contig 2). Features were drawn similarly to **(A)**. **(C)** Location of the fourth *C. metapsilosis ALS* gene (*CmALS2265*) on contig 6. This region was orthologous to *C. parapsilosis* contig 005504 and *C. orthopsilosis* chromosome 6 but contained approximately 16 kb of extra sequence. The region contained the reading frame for *CmIff61* (GenBank accession number MK205443) that encoded a 341-amino acid protein not present in the other species. Gene names were shown above other coding regions. *???* encoded a hypothetical protein (*CPAR2_601270*; *CORT_0F02270*) that was conserved in all three species, but shorter in *C. metapsilosis*. *MAP2* = *CPAR2_601280*, *CORT_0F02280*; *SBA1* = *CPAR2_601260*, *CORT_0F02260*; *MRP4* = *CPAR2_601250*, *CORT_0F02250*. Some genes in this region (*ALA1*, *MRP4*, *SBA1*) were reminiscent of those that are located near *CaALS5*, *CaALS1*, and *CaALS9* on *C. albicans* chromosome 6 (Zhao et al., 2003).

Three *C. parapsilosis* genome sequences (strains CDC317, CBS6318, GA1) were found in the National Center for Biotechnology Information (NCBI) database^7^. The annotated CDC317 sequence was also located in the *Candida* Genome Database^8^. BLAST using *C. albicans ALS* gene sequences as queries revealed five *ALS* genes in strain CDC317 (Figure 1). Four of the *ALS* genes were located on contig 006372 and transcribed in the same direction; the fifth gene was located on contig 006139 (Figure 1). The same five *ALS* genes were detected in strain CBS6318 and their arrangement appeared similar to strain CDC317. Only two *ALS* genes were apparent in the GA1 genome sequence: one was most similar to *CpALS4800* while the other most closely resembled *CpALS660*.

*ALS* genes in *C. orthopsilosis* were noted originally in the sequence of strain 90-125 that was available on the *Candida* Gene Order Browser (Maguire et al., 2013) and in the NCBI database. Because these sequences were incomplete due primarily to misassembled repeated sequence regions, we generated a new genome sequence using both Oxford Nanopore (long-read) and Illumina data (Lombardi et al., 2019). In collaboration with the laboratory of Arianna Tavanti (Pisa, Italy), we deduced the *ALS* genes and validated their sequence and variability. Strain 90-125 encoded three *C. orthopsilosis* genes (Figure 1). Two were contiguous on chromosome 3 and transcribed in the same direction while the third gene was on chromosome 2, reminiscent of the *ALS* gene arrangement in *C. parapsilosis*. Flanking regions of the genome were orthologous in each species. Genome sequences for two more *C. orthopsilosis* strains were available on the NCBI database: MCO456 and AY2. BLAST searches with *C. albicans* and *C. parapsilosis ALS* queries revealed multiple “hits,” however, the sequences were not assembled completely enough to determine an accurate number of *ALS* loci.

A genome sequence for *C. metapsilosis* was available in the NCBI database (accession CBZN0200000000) and was reported to encode a single *ALS* gene (Pryszcz et al., 2015). We generated a novel genome assembly for strain ATCC 96143 using the long-read and Illumina technologies. The Oxford Nanopore flowcell produced over 4.3 Gb of sequence from 670,717 reads. The minimum read length was 1,000 bp while the maximum was 89,846 bp. The mean read length was 6,476 bp. The majority of reads were 6–30 kb in length, with the longest at 89 kb. The genome assembly separated into diploid chromosomes and was deposited in GenBank as assembly PQNC00000000. A summary of computational analyses and characteristics of the resulting genome sequences was provided in Supplementary File S1.

BLAST using previously identified *ALS* genes (including those from *C. albicans*, *C. parapsilosis*, and *C. orthopsilosis*) revealed four *ALS* genes in *C. metapsilosis* (Figure 1). Three were orthologs of the *C. orthopsilosis ALS* genes, reflecting the close relationship between the species (Tavanti et al., 2005). The fourth *C. metapsilosis ALS* gene did not have an ortholog in either *C. parapsilosis* or *C. orthopsilosis*. Figure 1 shows the corresponding genome region where the fourth *C. metapsilosis ALS* gene was located and insertion of a 16-kb DNA fragment that was not present in either *C. parapsilosis* or *C. orthopsilosis*. BLAST of the published *C. metapsilosis* genome assembly (CBZN0200000000) showed the same four genes as detected in the new ATCC 96143 assembly.

The 20 diploid contigs for the new *C. metapsilosis* ATCC 96143 genome assembly were arranged in descending order by size; similar data were examined for the *C. orthopsilosis* and *C. parapsilosis* genomes (Table 2). Color coding indicated the location of *ALS* genes from Figure 1A (red), Figure 1B (blue), and Figure 1C (purple). Variable chromosomal localizations for the *ALS* genes suggested the potential for large karyotypic differences between these three closely related species. For example, the *ALS* gene cluster was located on chromosome 3 in *C. orthopsilosis*, and on the fourth-largest contig in *C. parapsilosis*. Although *CpALS0660* and *CoALS800* appear to be orthologs, one is found on the sixth-largest contig in *C. parapsilosis* and on the second-largest in *C. orthopsilosis*. Karyotypes for these species were not apparent in the literature, nor were tools such as a physical map that was essential for guiding assembly of the *C. albicans* genome (Chu et al., 1993).

**Table 2:** Chromosomes and contigs for the *C. parapsilosis*, *C. orthopsilosis*, and *C. metapsilosis* genome sequences.

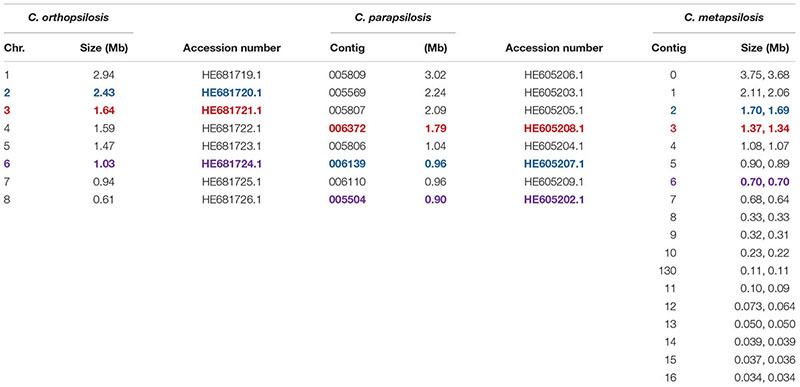

*Information was recorded for C. orthopsilosis 90-125 genome assembly ASM31587v1, C. parapsilosis CDC 317 genome assembly ASM18276v2, and the C. metapsilosis ATCC 96143 assembly PQNC00000000. Chromosome and contig sequences were ordered from largest to smallest within each species. C. orthopsilosis and C. parapsilosis were reported as haploid assemblies; diploid contigs were available for C. metapsilosis. Color coding indicated the location of the ALS gene cluster shown in Figure 1A (red), the single ALS gene shown in Figure 1B (blue), and the region encoding the unique CmALS2265 gene in Figure 1C (purple). Two C. metapsilosis contigs that encode mitochondrial genome sequences were not included in the table.*

Identification of new *ALS* genes raised the question of how they should be named. *ALS* genes in *C. albicans* are numbered starting with “1” and include *ALS1* through *ALS7*, and *ALS9* (Hoyer et al., 2008). Because the new genes presented here were different from the *C. albicans ALS* genes, we developed a new naming scheme based on Figure 1. The gene names (Table 3) reflected the close relationships between *ALS* genes in the *C. parapsilosis* species complex. The names accurately reflected the arrangement of genes on the various contigs, and the annotation available in the first genome assembly deposited in the NCBI database. For example, the names *CpALS4770* and *CpALS4780* suggested that the ORFs were *ALS* genes from *C. parapsilosis*. The consecutive numbers (4770 and 4780) were taken directly from the genome annotation and indicated that the genes were contiguous. Literature precedent for these ideas was already present (Table 3).

**Table 3:** *ALS* genes from the *Candida parapsilosis* species complex and their GenBank accession numbers.

		GenBank	Other
Gene	Size (bp)	Accession	References
***C. parapsilosis* strain CDC317**		
*CpALS4770*	3003	MH753532	*ALS1^a^, 404770^b^*
*CpALS4780*	3450	MH753533	*ALS5^a^, 404780^b^*
*CpALS4790*	7179	BK010629	*ALS3^a^, 404790^b^*
*CpALS4800*	4152	BK010630	*ALS4^a^, CpALS7^b^, CPAR2_404800^c^*
*CpALS660*	3072	MH753534	*ALS2^a^, 500660^b^*
***C. orthopsilosis* strain 90-125**		
*CoALS4210*	2457	MG799558	
*CoALS4220*	6078	MG799559	
*C0ALS800*	2499	MG799557	
***C. metapsilosis* strain ATCC 96143**		
*CmALS4210-1*	4722	MH753528	
*CmALS4210-2*	3267	MH753529	
*CmALS4220-1*	6714	MH753512	
*CmALS4220-2*	6837	MH753527	
*CmALS800*	3234	MH753530	
*CmALS2265*	6489	MH765692	

*Other references to the *ALS* genes were from ^a^Pryszcz et al., 2013; ^b^Neale et al., 2018; ^c^Bertini et al., 2016.*

Annotation of the *C. orthopsilosis* assembly proceeded somewhat differently than for *C. parapsilosis* (Riccombeni et al., 2012), leading to a different numbering scheme (Figure 1). We preserved the genome annotation tags in the names of the *ALS* genes (e.g., *CoALS800* came from *CORT_0B00800*). This scheme was carried forward to the gene names for the relatively unannotated *C. metapsilosis* genome assembly. Because the *C. orthopsilosis* and *C. metapsilosis* species were more closely related to each other than either was to *C. parapsilosis* (Tavanti et al., 2005), *C. metapsilosis ALS* gene names were matched to the *C. orthopsilosis* names. The name of the new gene, *CmALS2265*, reflected its location in the *C. metapsilosis* genome, while paying homage to the *C. orthopsilosis* assembly (i.e., it occupied the orthologous physical location between *CORT_0F02260* and *CORT_0F02270*).

### Als Protein Structure Encoded by the *C. parapsilosis* Species Complex *ALS* Genes

**FIGURE 2:**
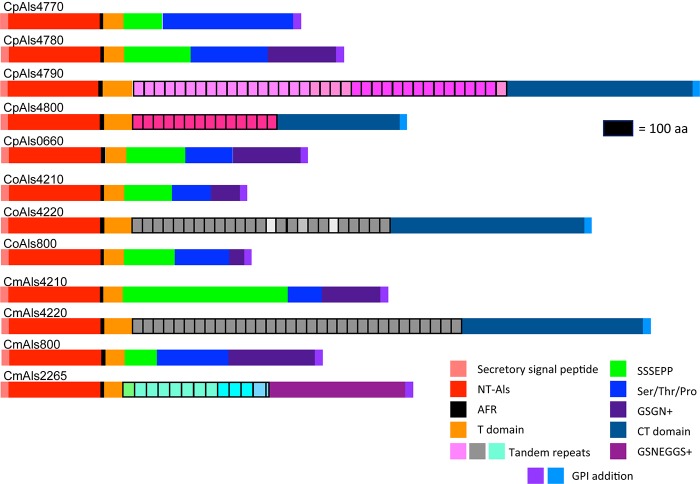
Schematic of proteins predicted from the *ALS* genes of the *C. parapsilosis* species complex. Each predicted protein had a secretory signal peptide, a classical NT-Als domain with eight conserved Cys residues to direct folding, an amyloid-forming region (AFR; Garcia et al., 2011), a Thr-rich sequence (T domain), a C-terminal domain rich in Ser/Thr, and a signal for addition of a GPI anchor that directs the mature protein to a final localization linked to β-1,6-glucan in the fungal cell wall (Lu et al., 1994). Only 5 of the 12 proteins included a central domain of tandemly repeated sequences like those found in *C. albicans* Als proteins (Hoyer et al., 2008). Different colors of the repeated units indicated differences in consensus sequence; shading within the same protein indicated repeat units that varied in the number of amino acids. The other proteins had regions of short, imperfect repeated sequences such as Ser-Ser-Ser-Glu-Pro-Pro (SSSEPP) and/or Gly-Ser-Gly-Asn (GSGN). CmAls2265 had the NT-Als domain attached to tandemly repeated sequences from the Iff/Hyr family (Bates et al., 2007; Boisramé et al., 2011) and a C-terminal region more-characteristic of Iff/Hyr proteins than *C. albicans* Als proteins.

*ALS* gene sequences were translated to amino acids, then used to draw a schematic of Als protein features (Figure 2). Color coding indicated similarities between the proteins. Visualization of the protein sequences provided additional insight into the gene arrangement displayed in Figure 1. For example, *CpALS4790* and *CpALS4800* each encoded proteins with a central tandem repeat domain, while proteins predicted by *CpALS4770* and *CpALS4780* were very similar to each other. These observations suggested that *CpALS4780* and *CpALS4800* arose by duplication of genes *CpALS4770* and *CpALS4790*, respectively. *CoALS4210* and *CmALS4210* were orthologs of *CpALS4770* while a similar relationship was evident for *CoALS4220*, *CmALS4220*, and *CpALS4790*. Proteins predicted from genes *CpALS0660*, *CoALS800*, and *CmALS800* were all very similar, consistent with the conserved genomic context for these ORFs.

Each predicted protein from the *C. parapsilosis* species complex (Figure 2) fulfilled various criteria for inclusion in the Als family. The proteins had a secretory signal peptide and a site for GPI anchor addition, consistent with a final localization in the fungal cell wall, linked to β-1,6-glucan (Lu et al., 1994). The high percentage of Ser and Thr in each protein, particularly in repeated sequences and toward the C-terminal end, also was consistent with composition of *C. albicans* Als proteins (Hoyer et al., 2008). Most importantly, each protein had an N-terminal domain that was similar to *C. albicans* NT-Als3 where adhesive function resides (Lin et al., 2014). NT-Als folding is driven by eight conserved Cys residues (Salgado et al., 2011), that were found in each predicted protein from the *C. parapsilosis* species complex.

**FIGURE 3:**
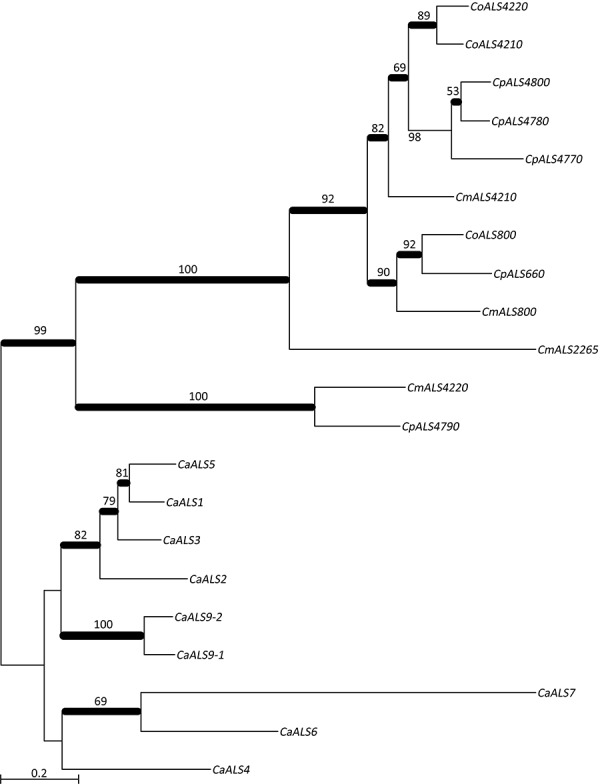
Phylogenetic tree of *ALS* sequences from *C. albicans* and the *C. parapsilosis* species complex. The tree was drawn using a ML analysis after removing ambiguous regions using Gblocks. Thickened branches indicated Bayesian posterior probabilities >95% while the numbers above the branches were bootstrap values >50%. The tree showed strong support for many branches.

NT-Als adhesive function is due to a PBC that can accommodate up to six C-terminal amino acids from peptide ligands in an extended conformation (reviewed in Hoyer and Cota, 2016). Als proteins recognize diverse peptide ligands with different Als proteins demonstrating higher-affinity interactions for specific sequences. Nucleotide sequences encoding NT-Als from the newly characterized genes were included in a phylogenetic analysis with the corresponding regions from the *C. albicans ALS* genes (Figure 3). *C. albicans* sequences grouped together in a manner that reflected known protein function (reviewed in Hoyer and Cota, 2016).

Based on positions of the genes in the phylogenetic tree, a few examples were selected for further examination of variation within the predicted PBC. Models of CpAls4790, CoAls4210, and CmAls2265 were created based on the structure of *C. albicans* Als9-2 (CaAls9-2) bound to the C-terminal residues of human fibrinogen gamma peptide (Protein Data Bank ID 2y7n; Salgado et al., 2011). For CaAls9-2, peptide binding is anchored by a salt bridge with an invariant Lys59 and further stabilized by main chain hydrogen bonding interactions residues S170, T293, and W295. The peptide has an extended conformation with side chains oriented alternatively on opposite sides of the peptide backbone similar to residues in a β strand. The amino acid side chains are inserted into conserved pockets of the protein. Amino acids lining the cavity are variable among known Als protein sequences, presumably modulating binding specificities among proteins in the Als family (Lin et al., 2014).

**FIGURE 4:**
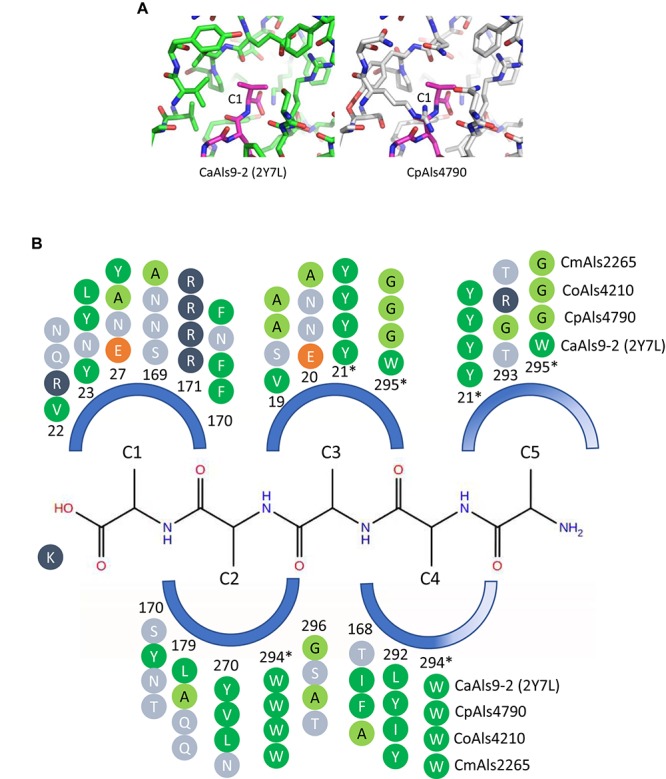
Structural comparison among peptide-binding cavities (PBCs) of a model Als protein and selected proteins from the *C. parapsilosis* species complex. **(A)** Side-by-side comparison of the C1 binding pocket of CaAls9-2 (left, green carbons; Protein Data Bank ID 2Y7N; Salgado et al., 2011) and a structural model of CpAls4790 (right, gray carbons) bound to fibrinogen gamma peptide (magenta carbons). The pocket was more constrained in CpAls4790 by the Val22Arg substitution, suggesting a more-selective binding activity than present in CaAls9-2. **(B)** Schematic of the NT-Als PBC showing the pockets surrounding amino acids of the bound peptide ligand (C1 through C5, with C1 proximal to the invariant Lys at the bottom of the PBC). Only residues which form the binding pocket were shown. Example proteins (CpAls4790, CoAls4210, CmAls2265) were selected from diverse parts of the phylogenetic tree in Figure 3. Side chain residues were indicated as circles and color-coded green (medium to large hydrophobic), light green (small hydrophobic), dark blue (positively charged), orange (negatively charged), or light blue (hydrophilic), and numbered to indicate their position within each protein. Residues with an asterisk were part of two pockets and the sequence was repeated for each pocket. The schematic highlights conserved positions (e.g., Y21, R171, W294) and illustrates variability found throughout the PBC.

Peptide-binding cavities for the new Als proteins were analyzed by sequence and structural comparisons (Figure 4A). Differences in size and electrostatic potential were assessed to compare peptide-binding capacities among the NT-Als proteins. The PBC was considered as multiple pockets (called C1 through C5) to facilitate this analysis. The sixth amino acid was not included in the analysis since the position was highly solvent-exposed in the CaAls9-2 structure.

There were large differences in PBC shape and charge among the proteins consistent with accommodation of peptides of various sequences. For example, the pocket surrounding the first C-terminal residue (proximal to the invariant Lys) in CaAls9-2 was formed by hydrophobic and hydrophilic residues suggesting the likelihood to accommodate a variety of amino acids. However, in CpAls4790, the pocket was much more constrained by substitution of CaAls9-2 Val22 with Arg in CpAls4790. The Arg residue filled a large part of the pocket and presented a positive charge directly adjacent to the C1 amino acid of the bound peptide. The substitution of Val22Arg in CpAls4790 suggested a more-selective ability to accommodate amino acids in the C1 position of the peptide sequence compared to CaAls9-2.

Further analysis of the first five C-terminal amino acids of the bound peptide identified the nearest residues within 8 Å of the β carbon of each amino acid in the peptide (or hypothetical β carbon in the case of glycine residues) which form the surface of the amino acid binding pocket. The sequence patterns of the C1 through C5 pockets showed variations in ability to accommodate peptide residues by both size and electrostatics (Figure 4B). Pocket C3 was highly constrained by close packing of neighboring residues allowing for accommodation of only very small or small residues like Gly, Ala, or Ser. Pockets C4 and C5 were partially solvent-exposed suggesting the likelihood of higher size promiscuity in accepting hydrophilic residues in these positions. Overall, the analysis was consistent with the conclusion that the *C. parapsilosis* species complex Als proteins possessed peptide-ligand binding activity similar to the highly characterized examples in *C. albicans* and, like the CaAls proteins, the newly identified proteins will demonstrate differences in selectivity for peptide binding.

**FIGURE 5:**
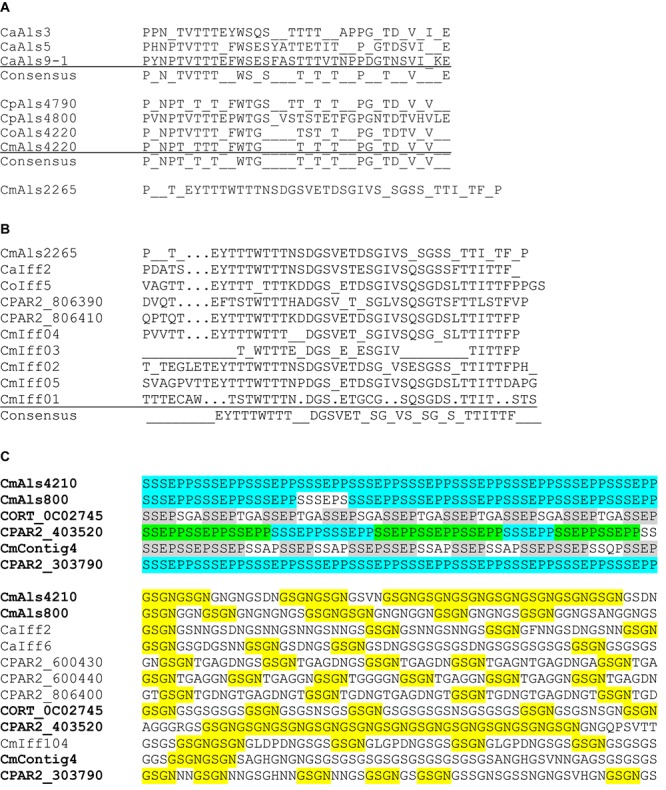
Conservation of tandemly repeated sequence units and short, imperfect repeats in various *C. albicans* and *C. parapsilosis* species complex proteins. **(A)** Consensus tandem repeat sequences were derived for *C. albicans* Als3 (CaAls3; GenBank accession number AY223552), CaAls5 (AY227440), and CaAls9-1 (AY269423). In *C. albicans*, each tandemly repeated unit was a perfect 36-aa length. Consensus sequences required that 80% or more of the amino acids were identical at each position. A similar approach was taken to aligning the consensus tandem repeat units from proteins from *C. parapsilosis*, *C. orthopsilosis*, and *C. metapsilosis*. GenBank accession numbers were shown in Table 3. In these proteins, the 36-amino acid repeat unit length was not necessarily conserved in each copy as color coded in Figure 2. The consensus repeat unit from CmAls2265 was 42 amino acids and different from the other Als proteins. **(B)** Alignment of consensus tandemly repeated sequence units from CmAls2265 and proteins from the Iff family. Repeat unit length was not necessarily conserved among the proteins, but a consensus sequence emerged from comparison among multiple proteins. GenBank accession numbers for the proteins were shown in Supplementary Table S4. **(C)** Top panel: *C. parapsilosis* species complex proteins that encode the short, imperfect SSSEPP (blue)/SSEPP (green)/SSEP (gray) repeated motif. Lower panel: Proteins that encode the GSGN (yellow) repeated motif. Protein identifiers were noted at the left of each sequence. The sequence labeled CmContig4 (GenBank accession number MK215077) most closely matched CORT_0E05950, which was annotated as Cdc24 GDP-GTP exchange factor, an unlikely identification of its function. Other proteins were labeled with identifiers from GenBank sequences or from the proteins listed in Supplementary Table S4. Names for proteins that had both SSEP/SSSEPP and GSGN short, imperfect repeats were listed in bold type in the top and lower panels.

Another common feature of *C. albicans* Als proteins is a central domain of multiple tandem copies of a 36-amino acid Ser/Thr-rich sequence. Previous thought had considered the *C. albicans* tandem repeats as grouping into three consensus sequences, represented by CaAls3, CaAls5, and CaAls9-2 (Hoyer et al., 2008). Although the absolute sequences are notably variable among different *C. albicans* Als proteins, a consensus sequence can be derived (Figure 5A). This consensus sequence was also found among proteins in the *C. parapsilosis* species complex tandemly repeated sequence units in the center of the protein (Figure 2). However, the repeated unit in CmAls2265 was clearly different in length and sequence from the other proteins. A BLAST search using the CmAls2265 repeat sequence as a query revealed the sequence in the *C. albicans* Iff family (Figure 5B) (Bates et al., 2007; Boisramé et al., 2011). This protein family was initially defined during annotation of the *C. albicans* genome sequence with “Iff” denoting Ipf family F, a group of proteins with similar sequence. The *C. albicans* Iff family also includes Hyr1 (Bailey et al., 1996). Subsequent investigations demonstrated various roles for these proteins, with a common feature being exposure on the cell surface (Boisramé et al., 2011). Supplementary Table S4 lists the currently recognized Iff proteins in *C. albicans*, as well as those revealed by BLAST searches of the *C. parapsilosis*, *C. orthopsilosis*, and *C. metapsilosis* genomes.

Analysis of the Iff family revealed its own consensus repeat sequence (Figure 5B) although the repeat was not found in each protein, and protein length varied considerably (Supplementary Table S4). A thorough description of the Iff family in *C. parapsilosis* and *C. orthopsilosis*, and comparison to the *C. albicans* Iff family was presented by Riccombeni et al. (2012) so will not be expanded upon here. Of highest importance to the current discussion was the idea that CmAls2265 was a hybrid protein, comprised of an NT-Als adhesive domain placed upon the “foundation” of an Iff protein. CmAls2265 was evidence of a recombination event between genes from two different cell wall-protein-encoding families as defined in *C. albicans*. The recombination event appeared specific to *C. metapsilosis*, as a similar gene was not found in either *C. parapsilosis* or *C. orthopsilosis*, highlighting another difference between the closely related species.

Some of the predicted Als proteins (Figure 2) had short, imperfect repeated sequences instead of the longer tandemly repeated units. Predominant sequence motifs in these proteins included SSSEPP, which tended to immediately follow the classical Als T domain, and GSGN, which was located more-proximal to the C-terminal end of the protein. These sequences were present in other cell-wall proteins from *C. albicans* and the *C. parapsilosis* species complex (Figure 5C). The GSGN motif was common among the Iff proteins including CaIff2, CaIff6, CPAR2_600430, CPAR2_600440, CPAR2_806400, and CmIff104. The SSSEPP motif was found in other proteins, such as CPAR2_303790, a hypothetical protein that also included the GSGN motif. The SSSEPP sequence sometimes was truncated (SSEPP or SSEP) and found in proteins that also included the GSGN sequences. One example was CORT_0C02745 (an Iff protein) and another was CPAR2_403520, which was annotated as a hypothetical protein with similarity to *C. albicans* Hwp1 (Staab et al., 1999). Sequence alignments between CPAR2_403520 and *C. albicans* Hwp1 did not produce convincing evidence of sequence conservation that suggested CPAR2_403520 had similar function to CaHwp1 (data not shown). The final protein that had both GSGN and SSEP was located on contig 4 of the new *C. metapsilosis* assembly and deposited in GenBank under accession number MK215077. The best match to this sequence was CORT_0E05950, currently annotated as Cdc24 GDP-GTP exchange factor. The presence of several sequences with the potential for such diverse function suggested more widespread domain swapping among proteins than indicated for CmALS2265. These observations also suggested the need for more-careful annotation of the sequences of the pathogenic fungi with respect to genes that encode cell-wall proteins with potential to interact with the host, other microbes, and the external environment.

### Allelic Variability Among the *C. parapsilosis* Complex *ALS* Genes

Allelic variability can be considered at multiple levels: the number of *ALS* genes in a specific fungal isolate is perhaps the most general. PCR was used to monitor *ALS* gene presence in additional strains of each species (Table 1). Primers were detailed in Supplementary Table S1. *C. parapsilosis* strains 949, 950, and 1125 each had the five known *ALS* genes, suggesting at least five *ALS* genes in each strain. Similar results were obtained for *C. orthopsilosis* and reported by Lombardi et al. (2019) where four different clinical isolates each encoded the three known *C. orthopsilosis ALS* genes. Analysis of *C. metapsilosis* isolates 61, 88, 397, and 482 indicated that, like the genome isolate ATCC 96143, each encoded four *ALS* genes.

*C. parapsilosis*, *C. orthopsilosis*, and *C. metapsilosis* are diploid, suggesting the potential for sequence and gene-length polymorphisms within each isolate. Current genome sequences for *C. parapsilosis* (CDC317; GenBank accession ASM18276v2) and *C. orthopsilosis* (90-125; ASM31587v1) are presented as haploid assemblies. These sequences were used as the basis for design of PCR primers to assess sequence and length polymorphisms among the *ALS* genes. Sequence polymorphisms were detected by Sanger sequencing of the PCR product. Length polymorphisms were visualized on agarose gels. This approach was effective at general validation of the published genome sequences although limitations must be recognized. For example, Sanger sequence reads were approximately 800-nt long, indicating the largest DNA fragment that could be assembled accurately was around 1.5 kb. PCR products larger than this size, or those that provided diploid allelic fragments of considerably different size were not amenable to this analysis. Accurate assignment of sequence polymorphisms to a specific allele would require construction of a heterozygous deletion mutant for each gene.

**FIGURE 6:**
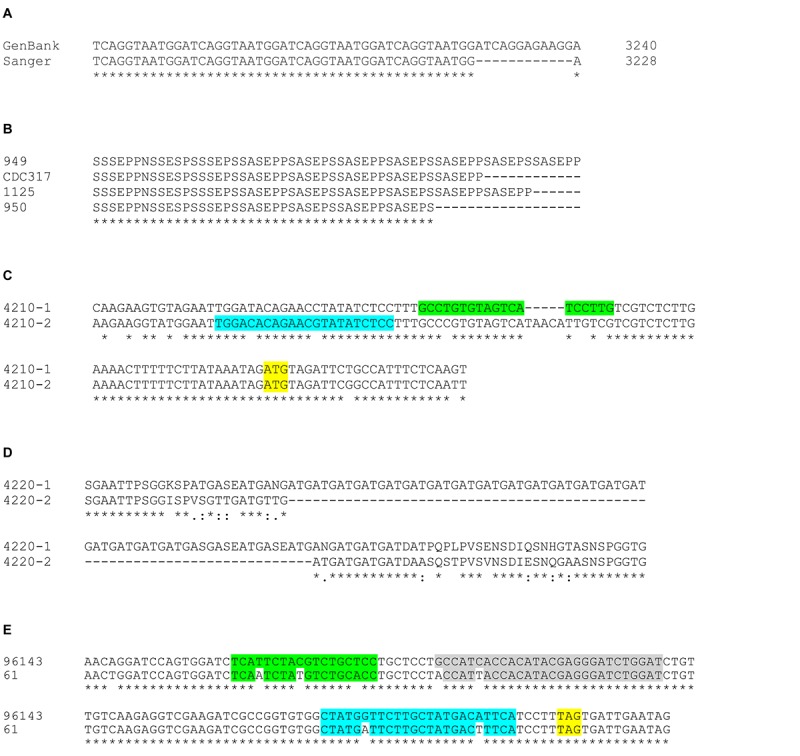
Examples of allelic sequence variation among *ALS* genes in the *C. parapsilosis* species complex. **(A)** The representative genome sequence for *C. parapsilosis* CDC317 (GenBank accession ASM18276v2) presented *ALS* genes in a highly accurate manner. Sanger sequencing in the current study revealed many sequence polymorphisms in the diploid *C. parapsilosis ALS* genes that were not reflected in the haploid GenBank assembly. However, there was only one notable example where Sanger sequence varied from the GenBank genome: *CpALS4780* had 12 fewer nucleotides in the region of the gene that encodes the GSGN short, imperfect repeat. **(B)** Regions of repeated sequences were often variable in length as shown in this example of *CpALS4770* alleles from various *C. parapsilosis* isolates (949, CDC317, 1125, 950). Manual alignment of the amino acid sequences underscored the different length of *CpALS4770* alleles in this region, leading to differing numbers of copies of the SSSEPP short, imperfect repeat. **(C)** The new *C. metapsilosis* genome assembly resolved into alleles and revealed previously unrecognized allelic variation that affected data interpretation. Primers to amplify *C. metapsilosis ALS* genes initially were designed from the representative genome sequence in GenBank (CBZN0200000000). The original forward primer to amplify *CmALS4210* (highlighted in green) amplified only allele 1 due to mismatches and extra nucleotides in the sequence for allele 2. Re-design of the primer (highlighted in blue) led to successful amplification of the *CmALS4210-2* allele, distinguishing it from *CmALS4210-1*. The *CmALS4210* start codon was highlighted in yellow. **(D)** Alignment between amino acid sequences predicted from the *CmALS4220* alleles. Considerable sequence variation between the alleles was observed including an expanded repeated sequence (Gly-Ala-Thr; GAT) in *CmAls4220-1*. These differences were predicted by the new *C. metapsilosis* genome sequence and verified by Sanger sequencing. **(E)** Allelic variation between strains of *C. metapsilosis* that affected interpretation of TaqMan assay data (see below). TaqMan assays were designed from the sequence of *C. metapsilosis* strain ATCC 96143. Nucleotide polymorphisms at the 3′ end of *CmALS2265* in strain 61 resulted in reduced detection by the TaqMan assay. The stop codon for *CmALS2265* was highlighted in yellow. The mismatches between the sequences for the forward primer (green), reverse primer (blue), and probe (gray) were sufficient to severely under-estimate relative gene expression for *CmALS2265* in strain 61, suggesting that the gene was barely transcribed.

Overall, however, these methods validated the high degree of accuracy of the CDC317 genome assembly. Because PCR products represented both alleles of the diploid species, sequence ambiguities were present in the Sanger data where there was variation in the allelic sequences. In general, sequence variation was greater toward the 3′ end of the gene, perhaps due to conservation of the sequence that encodes the NT-Als binding domain. Polymorphic nucleotides were less than 1% of the total sequence reads among the fragments meeting the criteria described above. In one instance, the CDC317 sequence diverged from our Sanger validation (Figure 6A), but only by 12 nucleotides in a region of short, imperfect repeats. Repeated DNA was the largest source of length variation between alleles in the same strain and between different strains. Allelic variation in the “classic” *ALS* repeat units was the most notable, with the potential to vary by as much as a few kb between alleles. Figure 6B shows an example of length variation within a region of short, imperfect repeats of CpALS4770 in five different *C. parapsilosis* isolates. Length polymorphisms between alleles in the same and different *C. orthopsilosis* isolates were documented clearly by Lombardi et al. (2019).

Advances in genome assembly software provide the tempting possibility to resolve allelic variation without the tedious and self-limiting validation steps described above. The newly derived *C. metapsilosis* ATCC 96143 genome assembly separated into allelic sequences. *CmALS800* was a complete, accurate sequence in both alleles, without any differences between the allelic nucleotide sequences. *CmALS800* was deposited in GenBank as a single allele (Table 3). In contrast, *CmALS4210* and *CmALS4220* each produced distinct allelic sequences in the new genome assembly that were deposited separately into GenBank (Table 3). Sequence polymorphisms were present throughout the coding region. In fact, sequence polymorphisms prevented *CmALS4210-2* from amplification with PCR primers designed against the published genome sequence (Figure 6C). Sequence differences within the region encoding NT-Als produced conservative changes unlikely to affect protein function. Length polymorphisms were most evident in regions of repeated sequences, with size estimates confirmed by agarose gel analysis of PCR products. The 3′ end of *CmALS4220-1* encoded expansion of a novel repeated sequence (Figure 6D).

*CmALS2265* was essentially identical in the genome assembly allelic sequences, although neither produced a complete open reading frame that encoded a full-length protein. The *CmALS2265* sequence had to be corrected by the PCR amplification and Sanger sequencing steps described above. To the extent possible using PCR amplification and Sanger data, the allelic sequences were validated as accurate in length and sequence. In one instance, nucleotide differences between *C. metapsilosis* strains affected use of a TaqMan assay designed to measure relative gene expression (Figure 6E; see below). Overall, these data provided evidence that the combination of short- and long-read sequence data and use of emerging genome assembly methodologies promise to more-accurately assemble genes that contain repeated sequences and resolve allelic variation in diploid species.

### Relative Expression Levels of *ALS* Genes in the *C. parapsilosis* Species Complex

One major goal of this work was to design and validate real-time PCR assays that can be used for quantification of relative *ALS* expression. Assay design was complicated by the high degree of similarity between *ALS* loci in the same species. TaqMan assays were used to exploit their ability to specifically differentiate between highly similar sequences. Initial assay design attempted to standardize the location of the assay in each gene by using the region approximately 800 to 1,000 nt after the start codon. Primer and probe sequences for each assay were shown in Supplementary Table S2. Initial work focused on *C. orthopsilosis* since it encoded only three *ALS* genes.

**FIGURE 7:**
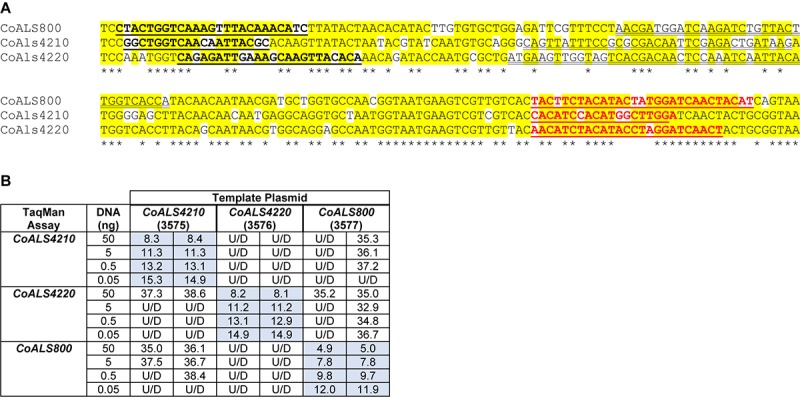
Demonstration of TaqMan assay specificity. TaqMan assays were selected for quantification of relative gene expression because of their exquisite specificity, which was needed to distinguish between highly similar loci in the same species. The most-extreme example was from *C. orthopsilosis* where sequences in the 5′ end of the gene were >75% identical. **(A)** Nucleotide sequences that were the most dissimilar in the 5′ end of *CoALS4210*, *CoALS4220*, and *CoALS800*. Yellow highlighting marked positions where at least two of the three sequences were identical. Asterisks marked the positions that were identical in all three sequences. In each sequence, the forward TaqMan primer sequence was underlined and shown in bold type. The TaqMan probe sequence was double underlined; each was in the sense orientation. The TaqMan reverse primer was underlined in bold, red type. **(B)** C_t_ values from TaqMan assays using cloned plasmid DNA as the reaction template. Dilutions of plasmid DNA were added to replicate TaqMan assays where the assay and template matched or were mismatched to gauge assay specificity. C_t_ values were recorded in the table and demonstrated recognition of the plasmid only when matched with the correct assay. For example, 5 ng of the cloned 5′ end of *CoALS4210* gave a C_t_ value of 11.3 when assayed with the *CoALS4210* TaqMan primers and probe. Undetectable signal (U/D) or a signal that was nearly undetectable (cycle 36.1) were recorded when the same amount of plasmid DNA was tested with the *CoALS4220* or *CoALS800* TaqMan assays. The data clearly demonstrated specificity of the TaqMan assays for their intended targets, even among genes as similar as the *ALS* genes of *C. orthopsilosis*.

*CoALS4210*, *CoALS4220*, and *CoALS800* were >75% identical in the targeted region (Figure 7A), providing the opportunity to demonstrate the ability of TaqMan assays to generate highly specific results. Dilutions of cloned target sequences were tested with the various assays and showed specific amplification only for matches between a given TaqMan assay and its intended target. Mismatched assay and targets were generally undetectable, even when adding large quantities of cloned plasmid to the reaction (Figure 7B).

**FIGURE 8:**
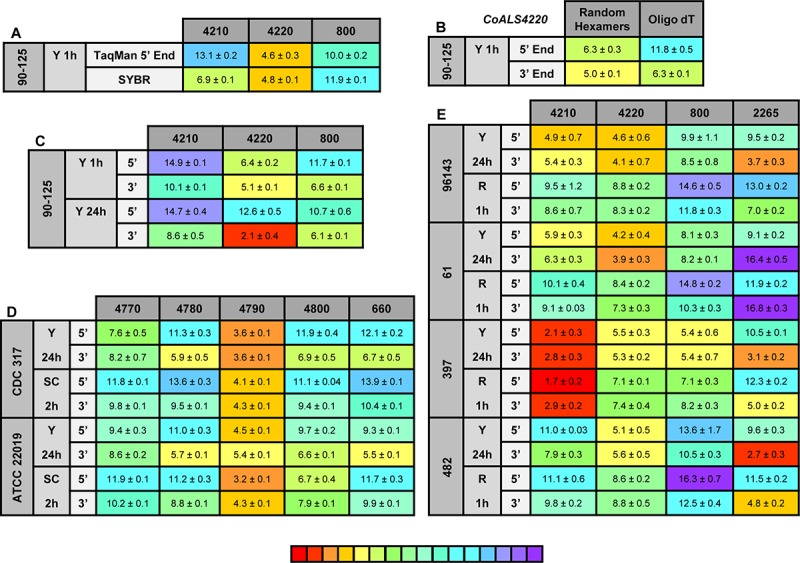
Relative gene expression measured by TaqMan assays. **(A)** *C. orthopsilosis* strain 90-125 was grown in YPD for 1 h. RNA preparations from these cells were assayed by TaqMan or the SYBR Green method of Lombardi et al. (2019). Mean (± standard error of the mean) C_t_ values were reported. Results were shaded to indicate the intensity of relative gene expression. The color key at the bottom of the diagram was shaded from red (high relative gene expression) to purple (low relative gene expression) to facilitate comparison between results. *CoALS4220* was expressed more highly than either of the other genes in the growth conditions tested. The SYBR Green method suggested a higher relative expression level for *CoALS4210* than did the TaqMan assay. **(B)** *CoALS4220* was used as a model gene to test the effect of cDNA synthesis priming method and TaqMan assay location on estimates of relative gene expression. Placement of the TaqMan assay at the 3′ end of the gene produced higher expression estimates than placement at the 5′ end of the gene. Priming with random hexamers produced a higher gene expression estimate than oligo dT priming. **(C)** Themes illustrated in **(A,B)** carried forward to a more-extensive analysis of *C. orthopsilosis ALS* gene expression. Strain 90-125 was grown for 1 h and 24 h in YPD medium. *CoALS4220* was more-highly expressed than the other genes. Estimates of relative gene expression were higher for TaqMan assays at the 3′ end of the gene than at the 5′ end of the gene. **(D)** Relative expression of *C. parapsilosis ALS* genes in two strains (CDC317, ATCC 22019) grown in two different conditions (YPD for 24 h; SC with fetal bovine serum for 2 h). *CpALS4790* was more-highly expressed than the other genes, even *CpALS4800* which was proposed to arise from duplication of *CpALS4790*. **(E)** Relative expression of *C. metapsilosis ALS* genes in four strains (ATCC 96143, 61, 397, 482) in two different growth conditions (YPD for 24 h; RPMI 1640 for 1 h). Considerable strain variation was observed with genes that were relatively quiet in one isolate (e.g., *CmALS4210* in strain 482) but extremely highly expressed in another (strain 397). In many instances, gene expression estimates from the 5′ end and 3′ end TaqMan assays provided similar results, although several notable exceptions were present. The apparent lack of *CmALS2265* expression in strain 61 (as measured with the 3′ end assay) prompted examination of the gene sequence at the site of the TaqMan assay (Figure 6E). Sequence variation between the TaqMan assay and gene sequence in this region explained the falsely low estimate.

Validated *CoALS* TaqMan results were compared to results from a previously reported SYBR Green-based assay (Lombardi et al., 2019). *C. orthopsilosis* strain 90-125 was grown in YPD Medium for 1 h and gene expression assessed using the SYBR Green and TaqMan assays. For SYBR Green, *CoALS4220* showed the highest relative expression, followed by *CoALS4210* (Figure 8A). The expression level for *CoALS800* was considerably lower than the other two genes. Results from the TaqMan assays showed the highest expression for *CoALS4220*, with *CoALS4210* and *CoALS800* at a similarly low expression level (Figure 8A).

One set of the SYBR Green assay primers (*CoALS800*) was located within the region to which the TaqMan assays were targeted while the others were located at the 3′ end of each gene (Lombardi et al., 2019). We used the gene with the highest expression level, *CoALS4220*, to test the effect of TaqMan assay location (5′ end vs. 3′ end of the gene), and method for priming cDNA synthesis (random hexamers vs. oligo dT). Since *CoALS4220* was 6078 bp long, we expected a low estimate of gene expression for the combination of the 5′ end assay and oligo dT priming of cDNA synthesis (Figure 8B). The relative gene expression from the combination of random hexamer priming and the 3′ end TaqMan assay was significantly higher than the other combinations (*P* < 0.0001). For each cDNA priming method, the 3′ end TaqMan assay gave a significantly higher gene expression estimate than the 5′ end assay (*P* = 0.002 for random hexamers; *P* < 0.0001 for oligo dT). Subsequent assays used random hexamer priming to make cDNA and both the 5′ and 3′ assays. The trend of lower gene expression (higher C_t_) estimates from the 5′ end assay continued in cells grown for 24 h in YPD medium (Figure 8C). *CoALS4220* was expressed more highly than the other genes in the two growth conditions studied (*P* < 0.0001).

Because of these results, both 5′ and 3′ end assays were developed for each of the 5 *ALS* genes in *C. parapsilosis* (Figure 8D). Two different strains were studied (genome strain CDC317, and ATCC 22019, the *C. parapsilosis* type strain). Cells were grown under two different conditions to dissect potential effects of strain, growth stage, and growth medium. One growth condition was suggested by the work of Lackey et al. (2013) who indicated that it promoted *C. parapsilosis* morphological change. *CpALS4790* was the most highly expressed gene in each growth condition, with similar estimates produced by the 5′ and 3′ end TaqMan assays. In many instances, the gene expression estimates from the 5′ end and 3′ end TaqMan assays were similar. Color coding in the image highlighted exceptions where the 3′ end assay provided as much as five cycles higher gene expression estimate than for the 5′ end assay. *CpALS4780* and *CpALS660* were more highly expressed at 24 h compared to 2 h, which could indicate a growth-stage or growth-medium effect on gene expression.

The same approach was used to assess relative *ALS* gene expression levels in 4 isolates of *C. metapsilosis* (Figure 8E) where gene expression differences between strains were more apparent than for the smaller sample of *C. parapsilosis* isolates. For example, *CmALS4210* expression was much higher in strain 397 than the others. The trend of higher gene expression estimates for the 3′ end assay was generally observed. One notable exception was *CmALS2265* in strain 61, for which the 3′ end assay produced considerably lower gene expression estimates than the 5′ end assay. PCR amplification of the assay site indicated that the 3′ end assay reverse primer sequence was not present in strain 61 due to sequence polymorphisms in that portion of the coding region (Figure 6E). Overall, these results demonstrated the complications with assay design to estimate relative gene expression and highlighted the need for sequence verification prior to studying gene expression in a variety of isolates.

## Discussion

The increasingly common availability of draft genome sequences for microbial pathogens is a tremendous benefit to the scientific community. Insight into genome composition promotes development of testable hypotheses regarding host-pathogen interaction. Genes in the *ALS* family have proven difficult to assemble because each fungal species encodes multiple, highly similar loci, and the loci tend to have extensive tracts of repeated DNA sequences (Hoyer et al., 2008); these repeated sequence units are similar in size to the read lengths from second-generation DNA sequencers posing a difficult assembly problem. Therefore, it is not surprising that genome assemblies frequently break within *ALS* coding regions or genes are misassembled with the 5′ end from one locus computationally attached to the 3′ end of a gene from a different physical location. The goal of this work was to develop an accurate description of the *ALS* family in each species within the *C. parapsilosis* species complex.

*ALS* gene sequences were well-assembled in the *C. parapsilosis* representative genome (CDC317) that was generated from whole-genome shotgun sequencing and available in GenBank. PCR amplification and Sanger DNA sequencing of *C. parapsilosis ALS* genes revealed numerous instances of nucleotide polymorphisms that are not specified in the public, haploid genome assembly, but only identified one instance where the genome assembly sequence diverged from the Sanger verification (Figure 6). In contrast, *ALS* genes were not completely assembled in the *C. orthopsilosis* representative genome (Co90-125), with gaps located in the repeated sequences. This observation prompted generation of a new Co90-125 sequence derived from a combination of long- and short-read sequence data (Lombardi et al., 2019). The effort aided completion of the three *ALS* gene sequences in this *C. orthopsilosis* isolate. The *C. metapsilosis* representative genome sequence was assembled from primarily short-read data from more than one strain (Pryszcz et al., 2015). Despite the published claim of one *ALS* gene in this species, four *ALS* genes were detected in both the representative genome and a new sequence generated from long- and short-read data from strain ATCC 96143. Overall, availability of long-read sequence data facilitated accurate *ALS* gene assembly although Sanger sequencing of PCR products was still needed in some instances to correct premature stop codons and produce a complete open reading frame.

*C. albicans ALS* genes were named by numbering them in the order in which they were characterized (*ALS1* to *ALS7*, *ALS9*; Hoyer et al., 2008). This traditional approach to naming genes was initiated before *C. albicans* genome data were available. These *C. albicans* names set a precedent and provide ample potential for confusion if applied to *ALS* genes in other fungal species. Orthology could be used as a criterion for naming genes among the fungal species considered here (Maguire et al., 2013); genes sharing a common physical location in different species could be given the same name with the expectation of similar function. These ideas become complicated for the *ALS* family because most species have a different number of *ALS* genes, gene order is not conserved well among the fungal species of interest, and there is a tendency toward tandem duplications of *ALS* loci (Figure 1). Als proteins are adhesins, yet function promiscuously, so looking toward shared function as the means for assigning names is also not an effective method. Some authors have referred to *ALS* genes using the label assigned in the annotation of the representative genome assembly (Bertini et al., 2016; Neale et al., 2018; Zoppo et al., 2018). This appealing approach provides a unique name for each gene and, like a street address, communicates physical location information. For example, it is obvious that *CPAR2_404770*, *CPAR2_404780*, *CPAR2_404790*, and *CPAR2_404800* are contiguous in the *C. parapsilosis* genome. The final gene names proposed here (*CpALS4770*, *CpALS4780*, *CpALS4790*, and *CpALS4800*) included physical location information from the genome annotation labels, an abbreviation to indicate species of origin, and “*ALS*” to signal inclusion in the gene family. A similar method was used to name the genes in *C. orthopsilosis* and *C. metapsilosis*.

Hoyer and Cota (2016) used “NT/T/TR/CT” to describe the standard composition of Als proteins in *C. albicans* where NT is the NT-Als domain that contains the PBC, T is the Thr-rich region, TR denotes the tandem repeats of the consensus 36-amino-acid sequence (Figure 2), and CT is the Ser/Thr-rich C-terminal domain. *C. albicans ALS* genes were used as BLAST queries to pull sequences from the *C. parapsilosis* species complex genomes. However, only four of the resulting Als protein sequences fit the ideal *C. albicans* definition. Seven of the predicted proteins lacked the TR domain and in the last protein, the TR domain was from a different family as defined in *C. albicans*. Other predicted proteins that lacked the TR domain instead encoded regions with short, imperfect repeats. Finding these short, imperfect repeats in other cell-surface proteins suggests additional recombination between *ALS* sequences and others. To incorporate the new *C. parapsilosis* species complex proteins into the Als family, the definition must shift away from NT/T/TR/CT. Instead, the Als family appears to include proteins that have an Als adhesive domain presented on a structure that promotes cell-surface display. This alternative Als family definition encompasses all proteins identified in this study and designates CmAls2265 as an Als protein, rather than an Iff/Hyr protein. Indeed, several of the genes discussed here have been tested for their contributions to cell adhesion. Bertini et al. (2016) showed that deletion of *CpALS4800* resulted in a *C. parapsilosis* strain with reduced adhesion to human buccal epithelial cells. Neale et al. (2018) documented a role for CpAls4800 in adhesion of *C. parapsilosis* to extracellular matrix proteins in a microfluidics assay using physiological fluid shear conditions. Zoppo et al. (2018) deleted *CoALS4210* and showed decreased adhesion to human buccal epithelial cells for the resulting *C. orthopsilosis* mutant strain. Overall, data presented here suggest that definitions of gene families established in *C. albicans* will need to be modified to accommodate data from other fungi.

Extensive analyses of *C. albicans ALS* gene expression revealed differential expression regulated by morphological form, growth medium, and stage of culture growth (reviewed in Hoyer et al., 2008). This information provided the foundation to assess Als protein function *in vitro*. TaqMan assays specific for individual *ALS* genes were developed to collect similar data for *ALS* genes in the *C. parapsilosis* species complex. Since *ALS* genes are large, assays were placed at a similar position within each coding region to avoid effects of assay location on gene expression estimates. Initial assays were placed within the region that encodes the NT-Als domain; a second set of assays was designed at the 3′ end of each gene. Surprisingly, results from the 5′ end and 3′ end assays frequently varied by several C_t_ units with the tendency toward higher expression estimates from the 3′ end assays. Since the TaqMan assays quantify cDNA as a reflection of RNA abundance, results showed that cDNA from the 3′ end of the genes is more abundant than cDNA from the 5′ region of the gene. Several factors can be invoked to explain this imbalance between cDNA abundance including the 3′ end of the transcript being more accessible to reverse transcription than the 5′ end, selective degradation of the 5′ end of the transcript, or the presence of another promoter that overlaps with the 3′ end of the transcript and boosts its relative abundance. Examination of the genome sequence did not provide obvious evidence for the presence of an overlapping ORF that could result in greater abundance of 3′ end transcripts. Use of the TaqMan assays to compare gene expression across several isolates of the same species revealed sequence heterogeneity that interfered with assay function in certain strains. Lack of target recognition could also partially explain the relative gene expression estimate disparities discussed above. For example, sequence variation between alleles within the same strain could reduce relative estimates of gene expression if the assay recognizes only one of the alleles in the diploid species. Regardless of the issues, TaqMan data pointed to certain genes as being the most-highly expressed and indicated considerable strain variation in gene expression. The TaqMan assays are useful for gene expression analyses but must be validated with the specific strains to be tested. Using more than one assay per locus is also recommended to avoid potential misinterpretations as described above.

The literature contains a few reports regarding relative expression of *ALS* genes in the *C. parapsilosis* species complex. Comparisons of *C. orthopsilosis* TaqMan data to gene expression studies conducted in the laboratory of Dr. Arianna Tavanti (University of Pisa) were presented in the “Results” section. Results were not directly comparable, leading to questions of whether gene expression is influenced by local factors. One potential factor that is noted in the *Candida* literature is water quality, which despite modern purification systems, may vary widely and influence biological function of fungal cells (Nickerson et al., 2012). Neale et al. (2018) measured expression of the five *C. parapsilosis ALS* genes using a SYBR Green-based method. Differential expression of *CpALS4800* and *CpALS4780* was noted when cells were transferred from saturated YPD cultures to tissue culture medium for 3 h. Like the TaqMan data presented here, expression of *CpALS4790* was highest in the saturated YPD culture, with *CpALS4780* next-most abundant and others at a lower level. Collectively, the data highlight and prioritize research questions to pursue. *CpALS4790* may be at the top of this list because of its high relative expression that predicts an abundance of CpAls4790 on the cell surface. However, structural predictions suggest that ligand binding may be more selective than for some of the other Als proteins (Figure 4). Perhaps CpAls4790 serves a more-focused adhesive role than deduced for the *C. albicans* Als proteins for which function was studied in detail (reviewed in Hoyer and Cota, 2016).

Future studies should also pursue greater insights into *ALS* gene expression patterns within the *C. parapsilosis* species complex. The current study was intended to assess whether *ALS* gene expression displayed the same differential patterns as observed in *C. albicans* and to identify genes that may produce proteins most abundantly on the fungal cell surface. In *C. albicans*, *ALS* genes that always showed a low expression level produced protein at a level undetectable by immunolabeling with specific monoclonal antibodies (Zhao et al., 2011). Genes expressed at a high level produced Als protein that could be long-lived on the cell surface (Coleman et al., 2010). It is also important to consider that gene expression and protein production can vary between *in vitro* and *in vivo* environments, suggesting the need for *in vivo* work to best understand the contributions of Als protein function in the host (Coleman et al., 2012).

Data presented here also set up intriguing experiments to pursue in *C. metapsilosis*. For example, the previous report that claimed only one *ALS* gene in the species was perhaps accepted without question because *C. metapsilosis* does not readily display phenotypes *in vitro* that are associated with pathogenic species. *C. metapsilosis* is less adherent to human buccal epithelial cells than either *C. parapsilosis* or *C. orthopsilosis* and shows a lower fungal burden at early stages of a murine vaginal infection model (Bertini et al., 2013). However, *C. metapsilosis* has four *ALS* genes, some are expressed at high levels, and the predicted proteins have an NT-Als domain like those in *C. albicans*. This new information must be reconciled with the relative lack of an adhesive phenotype for *C. metapsilosis* when assayed *in vitro*. Overall, data presented here highlight key questions that remain to be answered regarding the Als proteins and demonstrate the hypothesis-generating power of accurate genome assemblies.

## Author Contributions

CF, AH, and LH designed the experiments. S-HO, BSm, BSt, AM, CF, AH, and LH conducted the experiments. All authors analyzed the data, and wrote and approved the manuscript.

## Conflict of Interest Statement

The authors declare that the research was conducted in the absence of any commercial or financial relationships that could be construed as a potential conflict of interest.

## Footnotes

1 https://blast.ncbi.nlm.nih.gov/Blast.cgi

2 http://www.cbs.dtu.dk/services/SignalP

3 http://mendel.imp.ac.at/sat/gpi/gpi_server.html

4 https://www.ebi.ac.uk/services

5 https://www.ebi.ac.uk/Tools/msa/clustalo

6 https://sites.duke.edu/vilgalyslab/rdna_primers_for_fungi

7 www.ncbi.nlm.nih.gov

8 www.candidagenome.org
