# Supplementary material for: Agglutinin-Like Sequence (ALS) Genes in the Candida parapsilosis Species Complex: Blurring the Boundaries Between Gene Families That Encode Cell-Wall Proteins
Source: Front Microbiol. 2019 Apr 26;10:781. doi: 10.3389/fmicb.2019.00781 (PMC6499006; doi:10.3389/fmicb.2019.00781)
Supplement: Supplementary file 1 [file Table_1.docx]

**TABLE S1** | Oligonucleotide primers.

| **Gene (GenBank Accession)** | **Primer** | **Sequence (5’-3’)** | **Location*** |
| --- | --- | --- | --- |
| *CpALS4770*  (MH753532) | CP4700NTF1 | CGGAAGGAAACTCAATATGGTCT | -118 to -96 |
|  | CP4700NTR1 | TAGTAGGAGTAATTGGGTCCCA | 1204 to 1225 |
|  | CP4770repeatF1 | TGGGACCCAATTACTCCTACTA | 1204 to 1225 |
|  | CP4770repeatR1 | GTTTGCTGGTGCGAATGAAG | 1706 to 1725 |
|  | CP4770CTF1 | CTTCATTCGCACCAGCAAAC | 1706 to 1725 |
|  | CP4770CTSqF1 | GCCAACTTCATCAGGGACTATC | 2427 to 2448 |
|  | CP4770R1 | GCAGTGGATCGATTTGACGA | +89 to +108 |
| *CpALS4780*  (MH753533) | CP4780F1 | CCAGCCATGCATCTAGCTATAAA | -95 to -73 |
|  | CP4780NTR1 | GATTGTCGTAGTCCATGTCGAG | 1149 to 1170 |
|  | CP4780repeatF1 | CTCGACATGGACTACGACAATC | 1149 to 1170 |
|  | CP4780repeatR1 | CAGTAGGAGTAGTTGTGGATTCTG | 2060 to 2083 |
|  | CP4780repeatF2 | CAGAATCCACAACTACTCCTACTG | 2060 to 2083 |
|  | CP4780repeatR2 | CGTTTCCATTGTTTCCACTGTT | 2713 to 2734 |
|  | CP4780repeatF3 | AACAGTGGAAACAATGGAAACG | 2713 to 2734 |
|  | CP4780repeatR3 | GAGGCTCTCTTTATACCTCAATGT | +87 to +110 |
| *CpALS4790*  (BK010629) | CP4790F1 | ACAACTTCCCGTGTGTTAGC | -85 to -66 |
|  | Cp4790NTSqR1 | GGGTAGTTTGGTGCGGC | 136 to 152 |
|  | CP4790NTSqF2 | CGTATCAAAATGGCGAGGC | 968 to 986 |
|  | CP4790NTR1 | TCTACAATGACAGTGGCTGTAC | 1187 to 1208 |
|  | CP4790repeatF1 | GTACAGCCACTGTCATTGTAGA | 1187 to 1208 |
|  | CP4790repeatR1 | GGGTTATGTGGCTCGTATATGT | 3578 to 3599 |
|  | CP4790repeatF2 | ACATATACGAGCCACATAACCC | 3578 to 3599 |
|  | CP4790repeatR2 | GTTGATGAGATGGAGGGATCAG | 5240 to 5261 |
|  | CP4790CTF1 | CTGATCCCTCCATCTCATCAAC | 5240 to 5261 |
|  | Cp4790CTSqR2 | GCTTGGCGCATGACTAGAAGC | 5461 to 5481 |
|  | Cp4790CTSqF1 | GATGCCAGTGCTATAACGTC | 6025 to 6044 |
|  | CP4790CTR1 | AACTAGATGCAACGATGAGACC | +68 to +89 |
| *CpALS4800*  (BK010630) | CP4800F1 | ACTCTCGAATTGTATCGAAGACC | -180 to -158 |
|  | Cp4800NTSqF2 | CCCAGAGGTGGCAGATGC | 756 to 773 |
|  | CP4800NTR1 | TCAACAACAACAGTAGCGGT | 1192 to 1211 |
|  | CP4800repeatF1 | ACCGCTACTGTTGTTGTTGA | 1192 to 1211 |
|  | CP4800repeatR1 | TGATGCATGAGTCGATCTTCG | 2902 to 2922 |
|  | CP4800CTF1 | CGAAGATCGACTCATGCATCA | 2902 to 2922 |
|  | Cp4800CTSqR1 | CAGCGGTGCTATCAACTTCTG | 3023 to 3043 |
|  | CP4800R1 | GTAGAACCGAAATTGACCAATGTAA | +49 to +74 |
| *CpALS660*  (MH753534) | CP500660F1 | GACATTGCTTGACGGCATTC | -108 to -89 |
|  | CP500660NTR1 | CTTTCCAGGTGTAGCTGTGAT | 1168 to 1188 |
|  | CP500660repeatF1 | ATCACAGCTACACCTGGAAAG | 1168 to 1188 |
|  | CP4780repeatR1 | CAGTAGGAGTAGTTGTGGATTCTG | 2003 to 2026 |
|  | CP500660repeatF2 | TTCCAACCCAACCACTCTG | 2247 to 2265 |
|  | CP500660R1 | GGCCCTCTCCAGTTTAAATCA | +65 to +88 |
| *CmALS4210*  (MH753528) | CmTig64NT-F1 | GCCTGTGTAGTCATCCTTG | -121 to -103 |
|  | CmTig64NT-F2 | TGGACACAGAACGTATATCTCC | -121 to -103 |
|  | CmTig64NT-R1 | GGCAGTTTCTCCCGGAG | 1181 to 1197 |
|  | CmTig64Sq-F1 | GGTTCTGGTGGGTCAG | 427 to 442 |
|  | CmTig64Sq-F2 | CTGGTGAGTTGGGGAGC | 955 to 971 |
|  | CmTig64Sq-R2 | GCAGTAGTTGATCCAAGCC | 1031 to 1049 |
|  | CmTig64TR2-F1 | CCATCTTCAAGCCCAGG | 3772 to 3788 |
|  | CmTig64TR2-R1 | GCCTGATCCGTTTCCTTTAC | 4541 to 4560 |
|  | CmTig64CT-F1 | GTAAAGGAAACGGATCAGGC | 4541 to 4560 |
|  | CmTig64CT-R1 | GAACTCTGCCCCAATCTC | +87 to +104 |
| *CmALS4220*  (MH753529) | CmTig64NT-F2 | GCAAAGGTATTTCCTCAACTG | -98 to -78 |
|  | CmTig64NT-R2 | GCACTTGTAGGATTGGTGATTG | 1274 to 1295 |
|  | CmTig64Sq-F5 | CAAGCATATGGGTGGGG | 949 to 965 |
|  | CmTig64Sq-R6 | CTCCTGACGAGAATGGC | 1059 to 1075 |
|  | CmTig64Sq-R7 | GAACAGTATCAGTACCACC | 1399 to 1417 |
|  | CmTig64TR-F2 | CAATCACCAATCCTACAAGTGC | 1274 to 1295 |
|  | CmTig64TR-R2 | GGAACTTCGACTATCACTGTATC | 4756 to 4778 |
|  | CmTig64CT-F2 | GATACAGTGATAGTCGAAGTTCC | 4756 to 4778 |
|  | CmTig64CT-R2 | GCAAATGTATGCAATAACCG | +76 to +95 |
|  | CmTig64Sq-F6 | CACAAACGTGGTCAGG | 4694 to 4709 |
|  | CmTig64Sq-F7 | CAAGTGATACTAGCGAGGC | 5255 to 5273 |
|  | CmTig64Sq-F8 | CAGTCTACTTTGGGCGC | 5923 to 5939 |
|  | CmTig64Sq-R8 | GAGAATGAGGTGGTGACGC | 5321 to 5369 |
|  | CmTig64Sq-R9 | CTAGGTTGCGACTCGG | 6002 to 6017 |
| *CmALS800*  (MH753530) | CmTig84NT-F | CTGCGAGCTCCCATTG | -97 to -82 |
|  | CmTig84NT-R | GAGCTGAGTGGGTCCC | 1208 to 1223 |
|  | CmTig84Sq-F1 | GGTGGTTCAGGAGGTGATG | 424 to 442 |
|  | CmTig84Sq-F2 | GGCACCATACGACAACG | 963 to 979 |
|  | CmTig84Sq-R2 | GTTGGGATTGGTACGTC | 1102 to 1118 |
|  | CmTig84TR1-F | GGGACCCACTCAGCTC | 1208 to 1223 |
|  | CmTig84TR1-R | GGAGCAGAAGATGGTTGG | 1629 to 1646 |
|  | CmTig84Gap-F | CCAACCATCTTCTGCTCC | 1629 to 1646 |
|  | CmTig84Gap-R | CCAGGTGTTCCACTTGG | 2260 to 2276 |
|  | CmTig84TR2-F | CCAAGTGGAACACCTGG | 2260 to 2276 |
|  | CmTig84TR2-R | GAGCCTGAGCCTGATCC | 3043 to 3059 |
|  | CmTig84CT-F | GGATCAGGCTCAGGCTC | 3043 to 3059 |
|  | CmTig84CT-R | GCATGAATACTGGCTGCG | +137 to +154 |
| *CmALS2265*  (MH765692) | CmTig84NT-F2 | GTTGGAAGGGTCGTAACCATTA | -106 to -85 |
|  | CmTig84Sq2-F1 | GGCTCCTATTGGAATGATGC | 541 to 560 |
|  | CmTig84NT-R2 | CTGTGACACTATTCCGGAGTCG | 1272 to 1293 |
|  | Cm-Gene4-TRSeqF3 | ACAATAACTGCCACACC | 1168 to 1184 |
|  | Cm-gene4-TR-F2 | CACCAATTCAAACTGAGTACACTAC | 1331 to 1355 |
|  | CmTig84TR1-R2 | CCAGGGGCAGTAGCAC | 5319 to 5339 |
|  | Cm-Gene4-CT-F2 | CACTACCACATGGACCACTAC | 5319 to 5339 |
|  | CmTig84TR2-F2 | GTGCTACTGCCCCTGG | 5753 to 5768 |
|  | Cm-Gene4-CTSeqR3 | AGATCCTGTTCCAGTAGG | 5836 to 5853 |
|  | Cm-gene4-CT-R2 | GCATCAACAACATCCACCTTTAT | +168 to +190 |
| *ACT1* | PanACT1F | GTAAAGCCGGTTTCGCCG | 4 to 21 |
|  | PanACT1R | GAGATCCACATTTGTTGGAAAGT | 1006 to 1028 |
| *TEF1* | PanTEF1F | ATGGGTAAAGAAAAAACTCACGT | 1 to 23 |
|  | PanTEF1R | CAGCCTTGGTAACCTTACC | 1336 to 1354 |
| *CoALS4210*  (MG799558) | 4210RtF | AGACCCCACTAGCCACTTCT | 2057 to 2076 |
|  | 4210RtR | GCCTGATCCATTTCCACCATT | 2113 to 2133 |
| *CoALS4220*  (MG799559) | 4220RtF | CATGGTGGACATTGTCACAAC | 5927 to 5947 |
|  | 4220RtR | GCCCGAACCTTCATAAGTGT | 5999 to 6018 |
| *CoALS800*  (MG799557) | 800RtF2 | GTGTGCTGGAGATTCGTTTC | 915 to 934 |
|  | 800RtR2 | ACTTCATTACCGTTGGCACC | 988 to 1007 |
| *CoACT1*  (CORT_0D01620) | OrthoACT1RtF | TTCCCAGGTATTGCTGAACG | 919 to 938 |
|  | OrthoACT1RtR | GGAAAGTGGACAATGAAGCC | 1041 to 1060 |

*Primer location was relative to the GenBank accession listed in the first column. Negative signs indicated primers that were upstream of the coding region while plus signs denoted primers downstream of the coding region. Sequences upstream and downstream of the coding region were located on Candida Genome Database ([www.candidagenome.org](http://www.candidagenome.org)) or from larger contigs in GenBank.
