# Supplementary material for: Agglutinin-Like Sequence (ALS) Genes in the Candida parapsilosis Species Complex: Blurring the Boundaries Between Gene Families That Encode Cell-Wall Proteins
Source: Front Microbiol. 2019 Apr 26;10:781. doi: 10.3389/fmicb.2019.00781 (PMC6499006; doi:10.3389/fmicb.2019.00781)
Supplement: Supplementary file 2 [file Table_2.docx]

**TABLE S2** | TaqMan assays for *C. parapsilosis*, *C. orthopsilosis*, and *C. metapsilosis* *ALS* genes.

Gene Forward Primer (5’ – 3’) Reverse Primer (5’ – 3’) Probe (5’ – 3’) Product PCR

(bp) Eff. (%)

| CpALS4770 | GGTAGAGACTATTTTAGCTACATCCC | CTGACAAGGACCTGTCAGT | CAACTTATACAAATAGATTTCAATGTGCAGGTCTGCTCAC | 194 | 92 |
| --- | --- | --- | --- | --- | --- |
| CpALS4780 | ATCTCGGCCTCAACCAAT | TTATTGATTGTGAGTTGTCACGT | AAATTACATAAACAGATATCAATGCGCGGGAGAAATTTT | 187 | 98 |
| CpALS4790 | CTTCGGATTATACTGTTACAAGTCTG | CCCGTATCCTTCTTCACCC | CAGCGTCGTGTGTTGCGGATACTAAGC | 200 | 97 |
| CpALS4800 | ATGCTATTTCTGCTTCTTCAAGT | ACAACCTTTGACTTGTCGC | ATAAATGTGTTGGCGAACAGCGCGC | 190 | 99 |
| CpALS660 | GCTATATCAGGCTCAACAAGC | CCCAAGTTGTAGATTTTGAACCA | CACATACTTGTGTGCAGGAAGTTCATCGGTTAA | 196 | 97 |
| CoALS4210 | GGCTGGTCAACAATTACGC | CCAAGCCATGTGGATGTG | CAGTTATTTCCGCGCGACAATTCGAGACTGATAAG | 162 | 101 |
| CoALS4220 | CAGAGATTGAAAGCAAGTTACACA | AGTTGATCCTAGGTATGTAGATGTT | ATGAAGTTGGTAGTCACGACAACTCCAAATCAATTACA | 162 | 97 |
| CoALS800 | CTACTGGTCAAAGTTTACAAACATC | ATGTAGTTGATCCATAGTATGTAGAAGTA | AACGATGGATCAAGATCTGTTACTTGGTCACCA | 174 | 98 |
| CmALS4210 | GGCTGCAGAAGCCATCA | AGTTGTCCTCAATTGTTGTCCA | TCAAGCTGTAATGGACGCTCTTACACAATCAAC | 138 | 98 |
| CmALS4220 | GTTACTCTTCAGACTACACCGT | CGTAACCTTAACTTGTTGGCC | CCGGCCTGTGTCGAGCTCTCAAGTA | 140 | 106 |
| CmALS800 | GCTGAAGCTATTTCTGCTTCT | TATGTTTGTAAGCTTTCACCTGG | TAGTTGCACTGAACAAGCATTTATGGTCAACTACAAC | 134 | 98 |
| CmALS2265 | GTGCAGATAAATTTTCCTTCACG | GACGTCATTGCTAGCTCCT | AACATGTAACTCTAGGGGATTAACCGTAACTTACTCGA | 134 | 107 |
| CpALS4770End | AAGCAAGCAAGCAACGAATA | TGCGTCTCTAAACCTAATTCATAAC | AAATGGAGCTGGCATGATTGGATTGGGA | 140 | 107 |
| CpALS4780End | TAACGAATCCGGTGAATCATTG | AGGTGGAGACTCTTTGAATAGAT | ACCGATACTACTTCTCCTGCAGTTTCAAGCT | 104 | 103 |
| CpALS4790End | CACCTACTCATCAAACTACTTCTTC | GCATCAGGTCCACTTAAAGATT | TCCTACTGGTGCCAATACCGCTGTTACT | 145 | 101 |
| CpALS4800End | AGTTCAATCGATGATGCTGATG | GATGGTAATTGTGTTGAGAATGTTG | TGAAGGTGGACAAGGTACAGTTCCAGGT | 137 | 101 |
| CpALS660End | CACAACACCACCGATCATTT | CTGATAATGATGAATAAACCAGTCAAG | CCAACTATGAAGGATCAGGTTCAAGTCAAGGTGT | 90 | 97 |
| CoALS4210End2 | ACCCCACTAGCCACTTCT | GCCTGATCCATTTCCACCA | TCAGCTACACTTCCACACAGTGGTGGAA | 75 | 102 |
| CoALS4220End | CTTCATTGTCAGTTGGTGATGT | TGGTGAATTTGGTGATGATGTAG | TAACCAAGGCTTAGCTCCATCTGCTCCA | 149 | 96 |
| CoALS800End | GTGGAAACAACGGTGGAAAT | TCAAGAATGAAGATAAACCGTGAG | GCCTCCATC/ZEN/ACAATCACCCACAAACACC | 162 | 91 |
| CmALS4210End | GGTTCCGGAGAAACGTTGAT | AGACAATGATGAAAAGACCAGC | CCGCCACAACCAACTCCAATACTCCAACC | 127 | 99 |
| CmALS4220End | GAGGACGTTATCACAACAACAA | TTGGAACCATCATCACCTTTG | TCATCATCACAAGCATCTGTTGCACCTGT | 116 | 99 |
| CmALS800End | CTAACCCATCAGTATCACAACAAC | AACACCATAAGTGGATCCTGA | AAGCCAATCACCATCCACCAACACCAAC | 122 | 103 |
| CmALS2265End | TCATTCTACGTCTGCTCCTG | TGAATGTCATAGCAAGAACCATAG | ATCCAGATCCCTCGTATGTGGTGATGGC | 110 | 98 |
| CmACT1 | GGTAGACTTGACTGACTACTT | GATGAGGTTTGCATTTCTTGTTC | TCAGAACGTGGATACGGTTTCTCCACCA | 149 | 99 |
| CmTEF1 | AAGACCTTGTTGGAAGCTATTG | TTGATGATACCTGTTTCAACTCTAC | TTGAACCACCAACCAGACCAACTGACAA | 143 | 100 |
| CmCoCpACT1 | GCYTTGGCTCCATCBTCAAT | CCAGATTCGTCGTAYTCTTGTTT | TGTCTGGATYGGTGGTTCYATCTTGGCT | 143 | 100 |
| CmCoCpTEF1 | GGTACTTCTCAAGCTGATTGTG | CATTTGACTGAGTCCATCTTGTT | ACCAGAGAACACGCTTTGTTGGCTTACA | 167 | 100 |
