# Supplementary material for: Agglutinin-Like Sequence (ALS) Genes in the Candida parapsilosis Species Complex: Blurring the Boundaries Between Gene Families That Encode Cell-Wall Proteins
Source: Front Microbiol. 2019 Apr 26;10:781. doi: 10.3389/fmicb.2019.00781 (PMC6499006; doi:10.3389/fmicb.2019.00781)
Supplement: Supplementary file 3 [file Table_3.docx]

**TABLE S3** | List of cloned 5’ end fragments from *C. parapsilosis*, *C. orthopsilosis*, and *C. metapsilosis* *ALS* genes used as controls in TaqMan assays.

Strain Forward Forward Primer Reverse Reverse Primer

Gene ID Primer Name Sequence (5’-3’) Primer Name Sequence (5’-3’)

*CpALS4770* 3570 Cp4470NT-F1 CGGAAGGAAACTCAATATGGTCT Cp4470NT-R1 TAGTAGGAGTAATTGGGTCCCA

*CpALS4780* 3571 Cp4780F1 CCAGCCATGCATCTAGCTATAAA Cp4780NT-R1 GATTGTCGTAGTCCATGTCGAG

*CpALS4790* 3572 Cp4790-F1 ACAACTTCCCGTGTGTTAGC Cp4790NT-R1 TCTACAATGACAGTGGCTGTAC

*CpALS4800* 3573 Cp4800NT-F1 CCGTCAAGTCTCACCAACA Cp4800NT-F1 TCAACAACAACAGTAGCGGT

*CpALS660* 3574 Cp660F1 GACATTGCTTGACGGCATTC Cp660NT-R1 CTTTCCAGGTGTAGCTGTGAT

*CoALS4210* 3575 Co1-1 CAATTGTCACCCGAAAATTTTCC Co1-2 CTCTGATGAACTTGGTAGTTCCAG

*CoALS4220* 3576 Co2-1 GGGACACAGAAACTCTTTCCC Co2-2 GGTCAACGTAGTTGTCGAATGG

*CoALS800* 3577 Co3-1 TGTAGGGCTCAGTTAAGTTCAAAT Co3-2 CTGATGAACTTGGAGACTCTGAT

*CmALS4210* 3578 CmTig64NT-F1 GCCTGTGTAGTCATCCTTG CmTig64NT-R1 GGCAGTTTCTCCCGGAG

*CmALS4220* 3579 CmTig64NT-F2 GCAAAGGTATTTCCTCAACTG CmTig64NT-R2 GCACTTGTAGGATTGGTGATTG

*CmALS800* 3580 CmTig84NT-F CTGCGAGCTCCCATTG CmTig84NT-R GAGCTGAGTGGGTCCC

*CmALS2265* 3581 CmTig84NT-F2 GTTGGAAGGGTCGTAACCATTA CmTig84NT-R2 CTGTGACACTATTCCTGAGTCG
