# Supplementary material for: Agglutinin-Like Sequence (ALS) Genes in the Candida parapsilosis Species Complex: Blurring the Boundaries Between Gene Families That Encode Cell-Wall Proteins
Source: Front Microbiol. 2019 Apr 26;10:781. doi: 10.3389/fmicb.2019.00781 (PMC6499006; doi:10.3389/fmicb.2019.00781)
Supplement: Supplementary file 4 [file Table_4.docx]

**TABLE S4** | Iff/Hyr proteins in *C. albicans* and the *C. parapsilosis* species complex.

***C. albicans*** ***C. parapsilosis*** ***C. orthopsilosis*** ***C. metapsilosis***

Protein Size GenBank CGD Protein/CGD Size GenBank Protein Size GenBank Protein Size GenBank

(aa) (aa) (aa) (aa)

CaIff1 1562 Q5A5M7.3 C7_03290C_A CPAR2_106910 404 CCE40656.1 CORT_0C02740 706 CCG25649.1 CmIff01 752 MK205435

CaIff2 1249 Q59XA7.1 C5_00730W_A CPAR2_106920 438 CCE40657.1 CORT_0C02745 857 CCG25650.1 CmIff02 1471 MK205436

CaIff3 941 Q5A029.2 CR_03630W_A CPAR2_301290 439 CCE41140.1 CORT_0C02750 774 CCG25651.1 CmIff03 1432 MK205437

CaIff4 1526 Q5AAL9.1 CR_00610W_A CPAR2_301300 414 CCE41141.1 CmIff04 1551 MK205438

CaIff5 1308 Q5A1E0.1 C4_06550C_A CPAR2_301310 411 CCE41142.1 CmIff05 1468 MK205439

CaIff6 1085 Q59XL0.2 C2_09130C_A CPAR2_301320 419 CCE41143.1 CmIff31 437 MK205440

CaIff7 1225 Q5A849.1 CR_00760C_A CPAR2_301330 437 CCE41144.1 CmIff32 429 MK205441

CaIff8 714 Q59XB0.2 C5_00710W_A CPAR2_404110 410 CCE44608.1 CmIff51 1197 MK205442

CaIff9 941 Q5A6U1.2 CR_03880W_A CPAR2_404120 420 CCE44609.1 CmIff61 341 MK205443

CaIff10 ~1245 XP_717775.2 C3_00580W_A CPAR2_600430 1429 CCE39630.1 CmIff101 414 MK205444

CaIff11 511 XP_717774.1 C3_00600W_A CPAR2_600440 1158 CCE39631.1 CmIff102 442 MK205445

CaHyr1 919 Q5AL03.2 C1_13450W_A CPAR2_702650 418 CCE45252.1 CmIff103 441 MK205446

CPAR2_702660 PDG N/A CmIff104 1701 MK205447

CPAR2_806390 1640 CCE42090.1

CPAR2_806400 2273 CCE42091.1

CPAR2_806410 1671 CCE42092.1

CPAR2_806420 1714 CCE42093.1
