# Supplementary material for: Agglutinin-Like Sequence (ALS) Genes in the Candida parapsilosis Species Complex: Blurring the Boundaries Between Gene Families That Encode Cell-Wall Proteins
Source: Front Microbiol. 2019 Apr 26;10:781. doi: 10.3389/fmicb.2019.00781 (PMC6499006; doi:10.3389/fmicb.2019.00781)
Supplement: Supplementary file 5 [file Data_Sheet_1.docx]

# File S1. *C. metapsilosis* Genome Sequencing and Assembly Methods

# Schematic of assembly process:

**Library prep and sequencing**

For Oxford Nanopore long-read sequencing, 1 μg of genomic DNA was sheared in a gTube (Covaris, Woburn, MA) for 1 min at 6,000 rpm in a MiniSpin plus microcentrifuge (Eppendorf, Hauppauge, NY). The sheared DNA was converted into a shotgun library with the LSK-108 kit from Oxford Nanopore, following their manual. The library was sequenced on a SpotON R9.4 RevC flowcell for 48 h using a MinION MK 1B sequencer.

**Computational analyses**

All analyses were run on the Biocluster, a high-performance computing cluster deployed at the Carl R. Woese Institute for Genomic Biology (http://biocluster.igb.illinois.edu). Basic job submission scripts and relevant source code used in the analyses are present in a Github repository (<https://github.com/HPCBio/Hoyer-June-2018>).

The analyses steps summarized below describe the relevant input data used in each step. The specific job scripts present in the repository are those used on the cluster from March-June 2018 for the assembly and have settings relevant for the optimal deployment of each analysis step, for example utilizing a local scratch disk space for speeding up steps, utilizing a specific computational partition for memory-intensive steps, etc.

## **Base calling of Oxford Nanopore data**

Base calling was performed from the Oxford Nanopore FAST5 data using Albacore v2.3.1 (Oxford Nanopore) to generate FASTQ reads and a sequence summary file:

FastQC v. 0.11.8 (1) was used to further access quality scores and other attributes of the data sets.

| **Measure** | **Value** |
| --- | --- |
| Filename | c_metapsilosis.fastq.gz |
| File type | Conventional base calls |
| Encoding | Sanger / Illumina 1.9 |
| Total Sequences | 770574 |
| Sequences flagged as poor quality | 0 |
| Sequence length | 36-89529 |
| %GC | 37 |

The following figures were selected from the initial FastQC report.


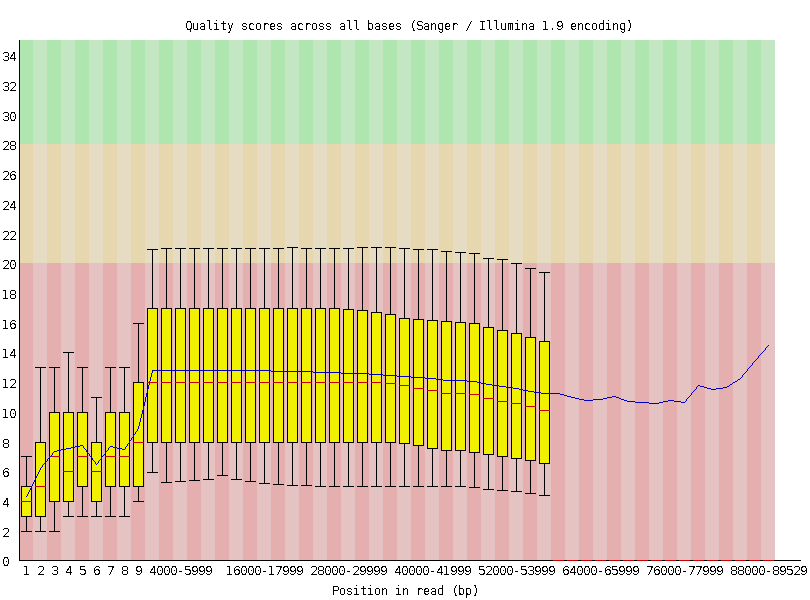


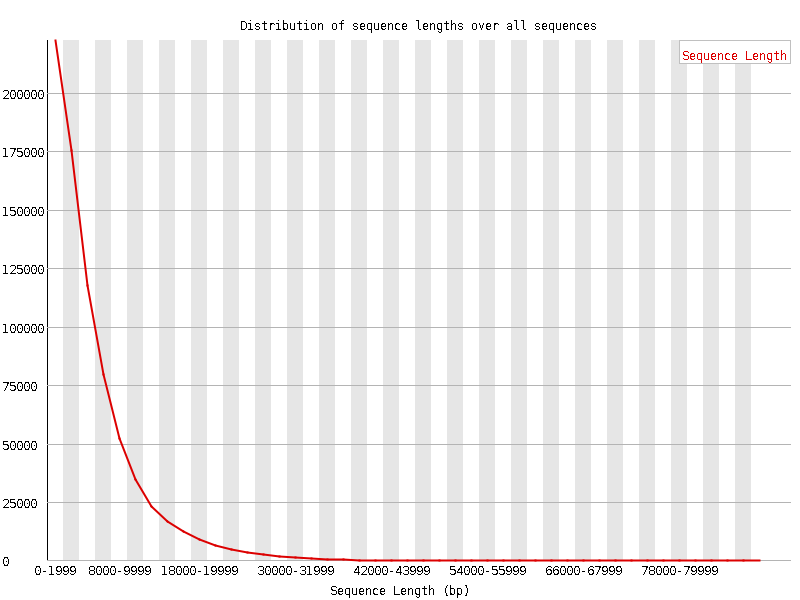


## **Barcode trimming – Oxford Nanopore**

Porechop (Github checkout 92c0b65f Feb. 2, 2018; <https://github.com/rrwick/Porechop>) was utilized as follows to remove barcodes and sequences with potential internal barcodes, followed by filtering reads using the tool seqtk v1.3 (<https://github.com/lh3/seqtk>) to remove any reads below 800-nt in length.

FASTQC was run on the resulting FASTQ file from Porechop:

| **Measure** | **Value** |
| --- | --- |
| Filename | c_metapsilosis.qualtrim.clean.fastq.gz |
| File type | Conventional base calls |
| Encoding | Sanger / Illumina 1.9 |
| Total Sequences | 685055 |
| Sequences flagged as poor quality | 0 |
| Sequence length | 800-89497 |
| %GC | 37 |


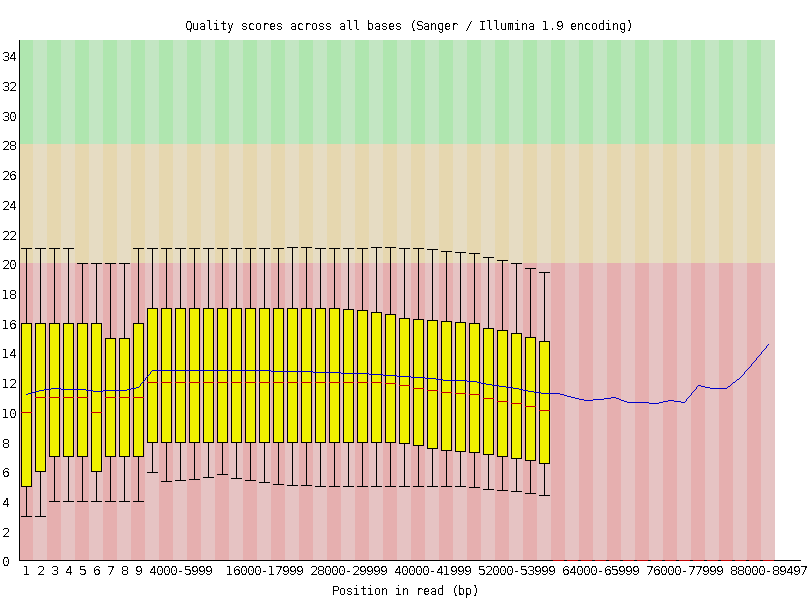


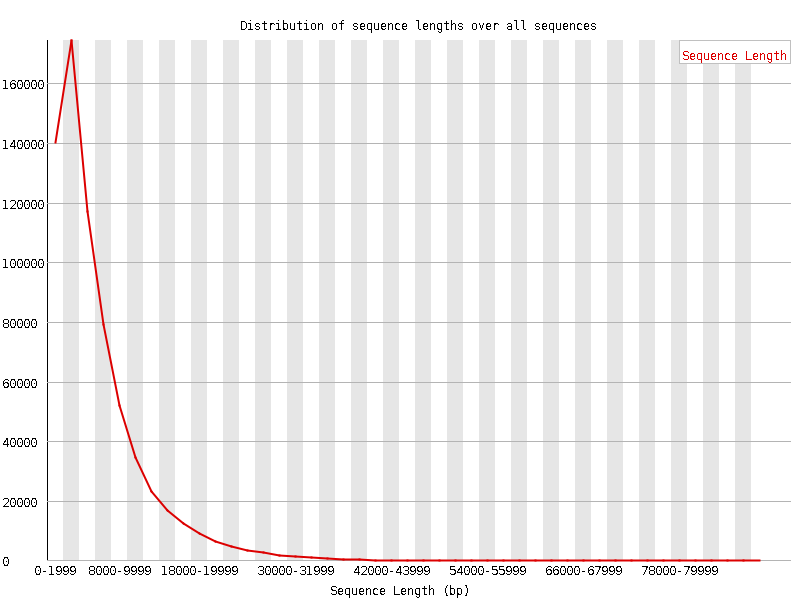


## **Quality trimming – Illumina MiSeq**

For the Illumina MiSeq reads, Trimmomatic-0.36 was used as follows to trim reads with quality scores less than 28 and remove reads with < 30 bp in length:

Raw and trimmed reads were all very high quality, and trimming removed very few reads.

| Filename | Total Sequences | Sequence length |
| --- | --- | --- |
| C_metapsilosis_ATTCCTTT_L001_R2_001.fastq.gz | 2,394,118 | 35-150 |
| C_metapsilosis_ATTCCTTT_L001_R1_001.fastq.gz | 2,394,118 | 35-150 |
| C_metapsilosis_ATTCCTTT.R1.qualtrim.paired.fastq.gz | 2,392,532 | 31-150 |
| C_metapsilosis_ATTCCTTT.R2.qualtrim.paired.fastq.gz | 2,392,532 | 30-150 |

### **Genome assembly**

ONP reads were assembled with Canu v1.7 (2) as follows:

The summary of the unpolished raw assembly results; the assembly size is notably larger than expected size of 13-14Mb, at 17.3Mb:

## **Initial polishing using Oxford Nanopore data**

Oxford Nanopore polishing utilized Nanopolish v. 0.9.0 (3) in several steps.

First, the original FAST5 data was indexed as follows:

Porechop-processed FASTQ data was aligned against the raw assembly using Minimap2 v2.8 (4), followed by sorting and indexing of the generated BAM file using Samtools v1.5 (5) as follows:

The sorted alignment and indexed FAST5 data were then used to polish the assembly using Nanopolish:

## **Final polishing using Illumina MiSeq data**

Trimmed Illumina MiSeq data were used in a Python script based on the iterative polishing step using in the assembler Unicycler (6), but assuming a diploid genome assembly. Reads are iteratively aligned to the Oxford-polished genome from Nanopolish using Bowtie v.2.3.2 (7); the resulting alignments are sorted and indexed, then polished using Pilon v1.22 (8). The resulting polished assembly is then used in the next iteration (re-align, re-sort, re-polishing) for up to 10 cycles.

After 6 cycles the number of corrections based on the resulting logged changes from Pilon reached a stable threshold but never seemed to reach zero. Upon further inspection, the remaining ‘corrections’ appeared to be from Pilon re-correcting some regions that may represent true haplotype differences. Based on this observation we chose to use the sixth iteration as the polished assembly, renaming the file as final.pilon.fasta and compressing the file using gzip. A separate step, Haplomerger2, was then utilized to resolve potential haplotype differences.


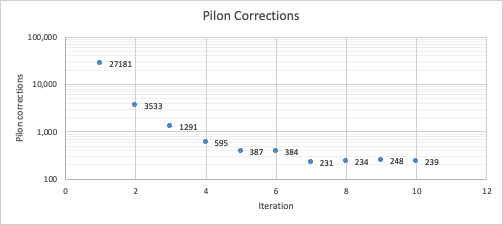


## **Resolving haplotypes**

A slightly modified version of Haplomerger2 v 20180603 (9) was utilized to locate potential haplotypes in the overall assembly and then resolve regions in the genome into a diploid assembly consisting of two phased haploid assemblies.

Haplomerger2 scripts were modified to run Haplomerger2 scripts from a base installation directory, utilizing the MiSeq-polished assembly as input. Each shell script was then simply called in succession with the input FASTA prefix:

The output from the final script were two haploid assemblies in FASTA format, the first considered the reference assembly and the second with the alternative haplotypes present. Scripts are present in the Github repository.

The final assembly metrics for the haploid reference assembly:

And the ‘alt’ haploid assembly:

## **Comparison of two phased genome assemblies**

A direct alignment of the two haploid files (reference vs alternate) was performed using MUMmer v4.0b2 (10):


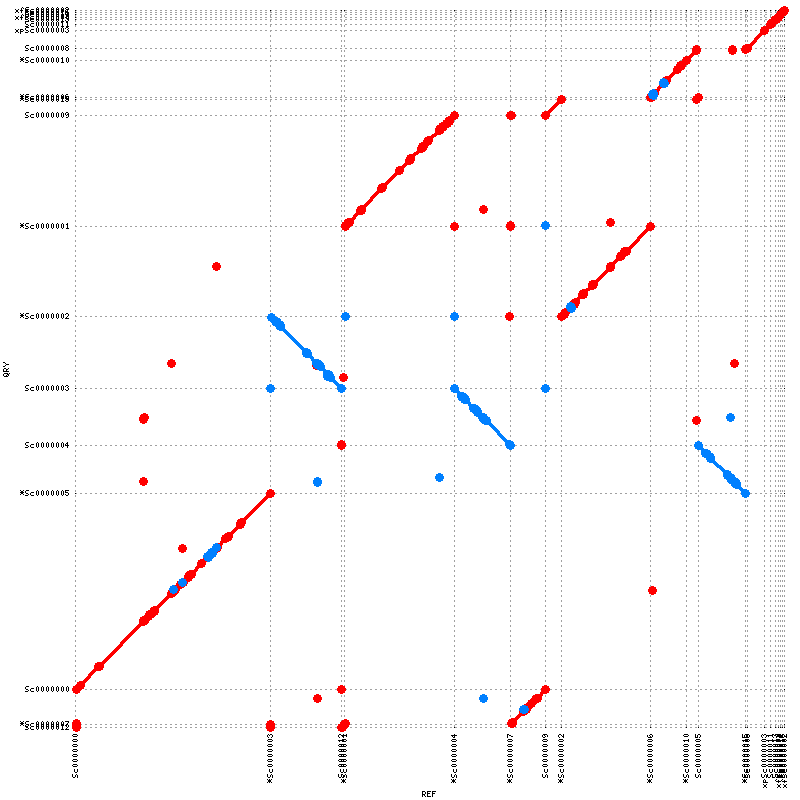


## **Comparison of final reference assembly to public assembly**

A comparison of the reference assembly vs the *C. metapsilosis* genome assembly (accessions CBZN020000001-CBZN020000096) from NCBI (<https://www.ncbi.nlm.nih.gov/Traces/wgs/CBZN02?display=contigs>):


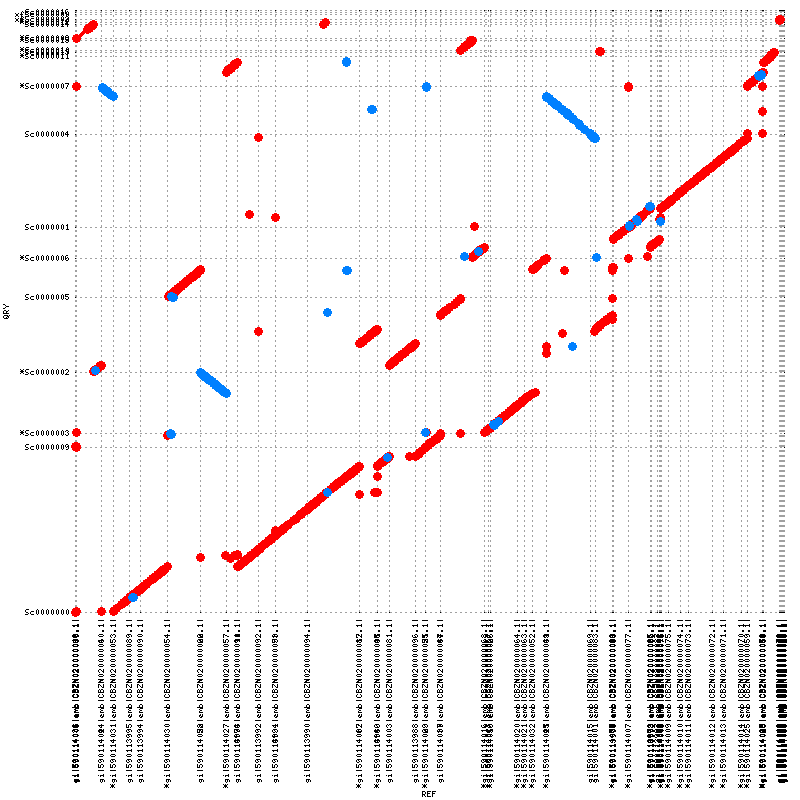


**References**

1. Andrews S (2016) FastQC: A quality control tool for high throughput sequence data.

2. Koren S*, et al.* (2017) Canu: scalable and accurate long-read assembly via adaptive k-mer weighting and repeat separation. *Genome Res* 27(5):722-736.

3. Simpson JT*, et al.* (2017) Detecting DNA cytosine methylation using nanopore sequencing. *Nat Methods* 14(4):407-410.

4. Li H (2018) Minimap2: pairwise alignment for nucleotide sequences. *Bioinformatics* 34(18):3094-3100.

5. Li H*, et al.* (2009) The Sequence Alignment/Map format and SAMtools. *Bioinformatics* 25(16):2078-2079.

6. Wick RR, Judd LM, Gorrie CL, & Holt KE (2017) Unicycler: Resolving bacterial genome assemblies from short and long sequencing reads. *PLoS Comput Biol* 13(6):e1005595.

7. Langmead B & Salzberg SL (2012) Fast gapped-read alignment with Bowtie 2. *Nat Methods* 9(4):357-359.

8. Walker BJ*, et al.* (2014) Pilon: an integrated tool for comprehensive microbial variant detection and genome assembly improvement. *PLoS One* 9(11):e112963.

9. Huang S, Kang M, & Xu A (2017) HaploMerger2: rebuilding both haploid sub-assemblies from high-heterozygosity diploid genome assembly. *Bioinformatics* 33(16):2577-2579.

10. Marcais G*, et al.* (2018) MUMmer4: A fast and versatile genome alignment system. *PLoS Comput Biol* 14(1):e1005944.
